# Formyl peptide receptor 1 agonism selectively suppresses mitochondria-driven neutrophil extracellular trap formation while preserving antibacterial immunity

**DOI:** 10.1186/s10020-026-01522-4

**Published:** 2026-06-13

**Authors:** Emil Bečka, Letícia Hudecová, Patrik Bertovič, Karolína Bíla, Ľudmila Podracká, Peter Celec, Michal Pastorek

**Affiliations:** 1 Institute of Molecular Biomedicine, Faculty of Medicine, Comenius University, Bratislava, Slovakia; 2 Department of Pediatrics, Faculty of Medicine, Comenius University and National Institute of Children’s Diseases, Bratislava, Slovakia; 3 Institute of Pathophysiology, Faculty of Medicine, Comenius University, Bratislava, Slovakia

**Keywords:** Neutrophil, Neutrophil extracellular traps, Mitochondria, Bacteria, DAMP, FPR1, TLR9, Surgery

## Abstract

**Background:**

Mitochondrial damage-associated molecular patterns (mtDAMPs) released after trauma can trigger neutrophil extracellular trap (NET) formation, potentially worsening tissue injury and increasing susceptibility to secondary infection. However, the mechanisms driving mitochondria-induced NETosis and whether this response can be selectively modulated without impairing antibacterial defense remain unclear.

**Methods:**

We investigated the mechanisms of mitochondria-induced NET formation using in vitro, in vivo models and ex vivo, using trauma patient plasma. Neutrophils were exposed to intact mitochondria or mitochondrial components, and the roles of Toll-like receptor 9 (TLR9), formyl peptide receptor 1 (FPR1), PAD4, NOX2, p38α, and STAT6 were assessed. Effect of the synthetic FPR1 agonist FPRA14 on NET formation and antibacterial neutrophil functions was tested in double hit in vivo model of peritoneal mitochondria injection followed by *S. aureus* pneumonia.

**Results:**

Intact mitochondria induced NET formation through coordinated activation of both TLR9 and FPR1, with dependence on PAD4, NOX2, and p38α signaling. Mitochondrial protein integrity was important for this response, likely by protecting mtDNA from degradation and enabling TLR9 activation. While insufficient to drive NETosis alone, FPR1 activation showed a concentration- and time-dependent modulatory effect: low-level activation promoted NETosis, whereas stronger or pre-exposure–induced signaling suppressed it through receptor desensitization. FPR1 agonist FPRA14 reduced mitochondria-driven NET formation in vitro and in a double hit murine model while preserving neutrophil antibacterial functions, including NET formation, phagocytosis, ROS generation, bacterial killing, and infection control. p38α acted as a positive regulator, whereas STAT6 functioned as a negative regulator of NET formation.

**Discussion:**

These findings identify FPR1 as a key regulator of pathological mitochondria-driven NETosis and suggest that selective FPR1 receptor desensitization may suppress excessive sterile inflammation without compromising host defense. This work extends current understanding of mtDAMP signaling in neutrophils and supports prophylactic FPR1 agonism as a potential strategy for limiting inflammatory tissue damage after trauma or surgery.

**Conclusion:**

Mitochondria-induced NETosis depends on TLR9/FPR1 signaling and can be reduced by FPR1 agonism without compromising antibacterial immunity. FPRA14 may represent a strategy to limit harmful sterile inflammation after trauma or surgery.

**Supplementary Information:**

The online version contains supplementary material available at https://doi.org/10.1186/s10020-026-01522-4.

## Introduction

Traumatic injury and major surgery release mitochondrial damage-associated molecular patterns (mtDAMPs) into the circulation, where their levels correlate with postoperative complications (Simmons et al. 2013; Zhao et al. 2014; Sandler et al. 2018). Owing to their bacterial endosymbiotic origin, mitochondria carry molecular signatures that mimic pathogen-associated molecular patterns such as unmethylated CpG DNA recognized by Toll-like receptor 9 (TLR9) and N-formyl peptides recognized by formyl peptide receptors (FPR1/2) (Hauser et al. 2010; Zhang et al. 2010). This enables neutrophils to sense tissue damage through the same pattern recognition receptors used for pathogen detection, triggering antimicrobial responses including neutrophil extracellular trap (NET) formation (Itagaki et al. 2015; Pastorek et al. 2023).

NETs trap bacteria, but simultaneously cause collateral tissue damage, perpetuate sterile inflammation and impair wound healing (Saffarzadeh et al. 2012; Brinkmann et al. 2004; Wong et al. 2015). This duality presents a challenge: inhibitory strategies targeting shared downstream effectors of NETosis (PAD4, gasdermin D, or DNase) cannot distinguish sterile from pathogen-induced response and risk attenuating host defense (Meng et al. 2012). Suppressing pathological NETosis while preserving antimicrobial immunity requires intervention at the receptor level, but the mechanisms of mtDAMP-induced NET formation remain poorly defined (Bečka et al. 2026).

A key gap in NETosis mechanistic studies is the predominant examination of individual purified mtDAMPs (mtDNA, ATP, succinate), whereas neutrophils in vivo encounter intact or partially degraded mitochondria, presenting multiple DAMPs simultaneously (Itagaki et al. 2015; Kim et al. 2020; Li et al. 2023). Whether whole mitochondria engage a single dominant receptor pathway or require coordinated multi-receptor signaling to induce NETosis is unknown. Formyl peptide receptor 1 (FPR1) is of particular interest because it activates multiple downstream signaling cascades, including calcium mobilization, MAPK pathways, and PI3K signaling, and undergoes rapid homologous desensitization upon sustained agonist exposure (Kaczmarek et al. 2018; Selvatici et al. 2006). Although FPR1 has been implicated in NET formation across diverse inflammatory contexts, mechanistic understanding of its role in NETosis is not complete (Bečka et al. 2026). Whether targeting FPR1 differentially affects sterile versus pathogen-driven NET formation is currently unknown.

Here, we characterized mechanisms by which whole mitochondria induce NETosis and tested potential of FPR1 agonism to selectively suppress it while preserving neutrophil antimicrobial activity. We demonstrate for the first time that mtDAMP-induced NET formation proceeds through coordinated TLR9–FPR1 engagement, requires protection of mtDNA within the organelle, and relies on multiple signalling nodes (PAD4/NOX2/p38). Critically, FPR1 agonism suppressed mitochondria-induced NET formation while fully preserving *S. aureus*-induced NETosis and bactericidal activity – both in vivo in a dual-hit model of surgical trauma followed by bacterial pneumonia, and in vitro in trauma patient plasma. Phosphokinase profiling confirmed p38 as a positive regulator and identified STAT6 as a novel negative regulator of mitochondria-induced NET formation downstream of FPR1 activation. Together, these findings establish FPR1-mediated signalling as a selective therapeutic target capable of dissociating sterile from pathogen-induced NET formation.

## Methods

### Human subjects

#### Clinical subjects

The study included 9 acute trauma patients, 4 healthy pediatric volunteers, and 6 healthy adult volunteers with respective sex and age listed in Table 1. None had prior medication use, immunosuppressive therapy, or oncological disease history.

Pediatric and trauma participants were recruited at the Department of Pediatrics and National Institute of Children's Diseases (Bratislava, Slovakia). Blood from trauma patients was collected within 1 h of admission. Ethics approval was obtained (Ethics Committee, University Hospital Bratislava, EK 218/2020), with written informed consent from all participants or guardians. No comorbidities were identified in any trauma individuals.

Healthy adult volunteers were free of acute or chronic inflammation at the time of sample collection. Separate ethical approval was granted for adult blood collection under the same ethics committee (project number EK 218/2020).

**Table 1:** Participant age and sex characteristics

Group	n	Mean Age ± SD (years)	Age Range (years)
Trauma patients	9		
Female	5	18.20 ± 2.28	16–22
Male	4	18.25 ± 1.50	16–19
Pediatric volunteers	4		15–23
Female	2	19.00 ± 5.66	15, 23
Male	2	17.50 ± 0.71	17, 18
Adult volunteers	6		
Female	3	26.00 ± 2.08	24–28
Male	3	31.00 ± 5.29	27–38

Plasma from the 9 trauma patients and 4 pediatric volunteers was used for transfer experiments. Whole blood and isolated neutrophils from healthy adult volunteers were utilized for all mechanistic, functional, and signaling studies.

### Blood collection and neutrophil isolation

Peripheral blood was drawn by venous puncture within 1 h of admission and collected into 10-ml BD Vacutainer® Heparin Tubes (367,886, Becton Dickinson, Plymouth, UK). Plasma was prepared by centrifugation at 1,600 × g, 10 min, 4 °C and stored at − 80 °C until use. Neutrophils were isolated from heparinized blood using 1-Step Polymorphs (Serumwerk) according to the manufacturer's protocol with previously described adaptations (Janko et al. 2023). Isolated neutrophils were resuspended in phenol red-free RPMI 1640 (Gibco, Paisley, UK) supplemented with 10% (v/v) heat-inactivated fetal bovine serum (FBS; PAN-Biotech, Aidenbach, Germany), counted on a LUNA-II automated cell counter (Logos Biosystems), and kept on ice until use.

## Reagents and biological materials

### Mitochondria isolation and processing

Human mitochondria (MITO) were isolated from fresh placental tissue (EK 218/2020) and mouse MITO were isolated from liver as previously described (Pastorek et al. 2023). Briefly, tissue aliquots (~ 100 mg) were homogenized in 1 ml ice-cold STE buffer (250 mM sucrose, 2 mM EGTA, 5 mM Tris–HCl, pH 7.4) using a Dounce homogenizer (30 strokes) and centrifuged at 500 × g, 3 min, 4 °C to pellet intact cells and debris. The supernatant was centrifuged at 8,000 × g, 10 min, 4 °C; the resulting pellet was resuspended in STE buffer and this wash centrifugation was repeated twice (3 cycles total). MITO were stored as dry pellets at − 20 °C. Particle number was quantified by flow cytometry. This procedure typically yields ~ 1 × 10⁹ particles per 100 mg of placental tissue. Mitochondrial purity and genome copy number were assessed by qPCR using primers targeting mitochondrial DNA (mtDNA) and nuclear DNA (ncDNA) listed in Table 2. qPCR was performed on a QTower3 cycler (Analytik Jena, Jena, Germany) using Advanced Universal SYBR Green Supermix (Bio-Rad, Hercules, CA, USA).

**Table 2:** Primer sequences

Organism	DNA origin and gene	Forward primer (5’−3’)	Reverse primer (5’−3’)
Homo Sapiens	**mtDNA** (D-loop hypervariable region)	CATAAAAACCCAATCCACATCA	GAGGGGTGGCTTTGGAGT
	**ncDNA** (human globin)	GCTTCTGACACAACTGTGTTCACTAGC	CACCAACTTCATCCACGTTCACC
Mus Musculus	**mtDNA** (cytochrome C)	CCCAGCTACTACCATCATTCAAGT	GATGGTTTGGGAGATTGGTTGATGT
	**ncDNA** (mouse globin)	TGTCAGATATGTCCTTCAGCAAGG	TGCTTAACTCTGCAGGCGTATG

For downstream processing, MITO aliquots were resuspended in assay-specific buffer. DNase-treated MITO were prepared by incubation with DNase I (0.04 KU/µl; 79,254, Qiagen, Hilden, Germany) for 30 min at 37 °C. Protein-depleted MITO were generated by incubation with Proteinase K (1 µg/µl; 3,115,836,001, Roche, Basel, Switzerland) for 1 h at 56 °C; both enzymes were inactivated at 70 °C for 10 min. Completeness of DNA and protein degradation was confirmed by 1.5% agarose gel electrophoresis and 10% SDS-PAGE, respectively (S1 Fig).

### Bacteria cultivation

*Staphylococcus aureus* (*S. aureus*) subsp. aureus Rosenbach (NCTC 8530) and *Escherichia coli* (*E.* coli) CFT073 (ATCC 700928) was grown overnight in LB broth (L3022, Sigma, St. Louis, MO, USA) at 37 °C with shaking (250 rpm). Bacteria were harvested at OD₆₀₀ = 1.0 (≈7.8 × 10⁸ CFU/ml, 8 × 10⁸ CFU/ml, respectively), centrifuged at 5,000 × g for 5 min, and resuspended in assay-specific buffer.

## In vitro neutrophil assays

### Live-cell imaging of NET formation

NET formation was quantified by live-cell imaging as previously described (Pastorek et al. 2023). Briefly, neutrophils (2 × 10^4^/well) were plated in 200 µl phenol red-free RPMI 1640 + 10% FBS (or 10% patient plasma for plasma stimulation experiments) in 96-well flat-bottom plates (83.3924.005, Sarstedt, Nümbrecht, Germany), stained with Hoechst 33,342 (1.25 µg/ml; Merck, Darmstadt, Germany) and SYTOX™ Green (200 nM; S7020, Invitrogen Waltham, MA, USA). Stimuli (~ 1 × 10^7^ particles of MITO or bacteria per reaction or as indicated in figures) were added immediately before imaging on a Cytation 7 multimode reader (BioTek, Winooski, VT, USA) using DAPI (Ex/Em: 377/447 nm) and GFP (Ex/Em: 469/525 nm) LED cubes with laser autofocus. Images were acquired every 10 min for 3 h for stimulus-induced NET experiments, or every 20 min for 4 h for patient plasma experiments, using a 10 × objective. Images were processed with rolling ball background subtraction; Hoechst 33,342⁺ objects (threshold 3,000; size > 5 µm) were counted as neutrophils, and SYTOX™ Green⁺ objects > 20 µm in diameter were scored as NETs to exclude debris. Relative NET formation was calculated as the AUC of total NET area over time, normalized to the initial neutrophil count (Hoechst 33,342⁺ objects at t = 0).

### Fluorescence microscopy

Neutrophils (1 × 10⁶/ml in RPMI + 10% FBS) were seeded onto poly-L-lysine-coated coverslips in 6-well plates and stimulated with vehicle or MITO (1 × 10⁷ particles) for 3 h at 37 °C/5% CO₂. Cells were fixed in 2% paraformaldehyde for 10 min, washed twice with PBS, and blocked with 5% BSA in PBS for 1 h. Coverslips were stained for 2 h with anti-MPO-FITC (1:500; ab11729, Cambridge, United Kingdom) and anti-LL37 (1:500; PA-LL37-100, Innovagen, Lund, Sweden) in blocking buffer, followed by donkey anti-rabbit PE (1:1000; 406,421, BioLegend, San Diego, CA, USA) secondary antibody staining. Coverslips were mounted with VectaShield Mounting Medium containing DAPI (H-1200–10, Vector Laboratories, Inc., Newark, CA, USA) and imaged on a Cytation 7 reader using DAPI (377/447 nm), GFP (469/525 nm), and RFP (555/584 nm) channels.

### Intracellular calcium measurement

Neutrophils (5 × 10⁶/ml) were resuspended in Ca^2^⁺/Mg^2^⁺-free HBSS and stained simultaneously with Fura Red (1 µM; F1242, Thermo Fisher, Waltham, MA, USA), FLUO-3 AM (1 µM; F3020, Thermo Fisher), anti-human CD66b-PE (305,106, BioLegend, San Diego, CA, USA), and Zombie NIR viability dye (1:1,000; 423,106, BioLegend) for 30 min at 37 °C together with the indicated pre-treatment (500 nM FPRA14). Intracellular Ca^2^⁺ kinetics were monitored by continuous flow cytometry acquisition for 300 s at 10 µl/min (DxFlex, Beckman Coulter, Brea, CA, USA); stimuli (1 µM fMLF, or ≈1 × 10^7^ particles of MITO or bacteria per reaction in 20 µl in HBSS) were added at t = 20 s, and 2 mM CaCl₂ was supplemented at t = 200 s to assess store-operated Ca^2^⁺ entry (SOCE). Intracellular Ca^2^⁺ is expressed as the FLUO-3/Fura Red fluorescence ratio in live (Zombie NIR⁻) CD66b⁺ neutrophils.

### FPR1 surface expression and internalization

Neutrophils (0.5 × 10⁶) were pre-treated with vehicle or 500 nM FPRA14 for 30 min at 37 °C and subsequently incubated with indicated stimuli (500 nM FPRA14, 1 µM fMLF, or ≈1 × 10^7^ particles of MITO or bacteria per reaction) in 200 µl RPMI for up to 20 min at 37 °C, then washed with ice-cold RPMI + 10% FBS + 2% BSA at 400 × g, 10 min, 4 °C. Cell pellets were resuspended in 100 µl of the same buffer and stained with anti-human FPR1-FITC antibody (2 µl; FAB3744F, Novus Biologicals, Centennial, CO, USA) and DRAQ7™ viability dye (600 nM; 424,001, BioLegend) for 60 min on ice in the dark, then immediately acquired on the flow cytometer.

### Phospho-kinase array

Neutrophils (5 × 10⁶) were resuspended in RPMI, pre-treated with vehicle or 500 nM FPRA14 and supplemented with indicated stimuli (≈1 × 10^7^ particles of MITO or bacteria per reaction) and incubated for 30 min at 37 °C. Cells were centrifuged at 400 × g, 10 min, 4 °C and lysed in the kit-provided lysis buffer supplemented with PMSF (100 µM) and protease inhibitor cocktail (1 ×; 539,131, Merck). Lysates were sonicated on ice and centrifuged at 20,000 × g, 10 min, 4 °C; supernatants were stored at − 80 °C. Phosphorylation profiles were determined using the Proteome Profiler Human Phospho-Kinase Array (ARY003C, R&D Systems, Minneapolis, MN, USA) according to the manufacturer's instructions. Data represent n = 3 independent experiments from separate donors.

### Neutrophil chemotaxis

Neutrophils (0.7 × 10⁶/ml in RPMI + 10% FBS) were pre-incubated with treatments for 30 or 15 min at 37 °C as indicated. Cell suspension (75 µl) was loaded into the upper chamber of a Multiscreen® 96-well polycarbonate membrane plate (8 µm pore; MAMIC3S10, Merck) and stimuli (100 nM fMLF or ≈1 × 10^7^ particles of MITO or bacteria in 150 µl) were placed in the lower chamber. After 90 min at 37 °C, migrated cells in the lower chamber were counted by flow cytometry.

### Phagocytosis assay

*S. aureus* and MITO were each adjusted to 1 × 10⁸ particles/ml in PBS. Bacteria were labeled with 0.5 µM BacLight™ Red Bacterial Stain (B35001, Invitrogen) and MITO with 100 nM MitoView™ Green (70,054, Biotium, Fremont, CA, USA) for 15 min at 37 °C in the dark. Labeled particles were washed once at 5,000 × g for 5 min and resuspended in RPMI 1640 + 10% FBS. Neutrophils (2 × 10^5^) were co-incubated with labeled particles at a 1:100 ratio (neutrophils:particles) in a total volume of 200 µl at 37 °C/5% CO₂. At the indicated time-points (10 and 30 min), samples were placed on ice to arrest phagocytosis and centrifuged at 400 × g for 10 min at 4 °C to pellet cells. The supernatant was collected and residual un-phagocytosed particles were quantified by flow cytometry. Phagocytic uptake was expressed as the percentage of particles depleted from the supernatant relative to a parallel cell-free control.

### Antimicrobial killing assay

Neutrophils (1 × 10⁶/ml in RPMI + 10% FBS) were pre-incubated with indicated stimuli for 30 min at 37 °C. *S. aureus* (~ 1 × 10^4^ CFU) was centrifuged at 5,000 × g for 5 min, resuspended in autologous serum to allow for opsonization, and co-incubated with neutrophils at a 10:1 volume ratio for 1 h at 37 °C/5% CO₂. Serial dilutions in PBS were spot-plated (10 µl) on LB-agar, incubated overnight at 37 °C, and CFU counted manually.

### Neutrophil ROS production assay

Neutrophils (2 × 10^5^/sample in RPMI + 10% FBS) were incubated with the indicated pre-treatment for 30 min at 37 °C. Then, stimuli (MITO, *S. aureus*) were added at a 1:100 ratio (neutrophils:stimuli) in a total volume of 200 µl at 37 °C/5% CO₂. Samples were concurrently stained with 1 µM Dihydrorodamine 123 (D632, Invitrogen) and Zombie NIR viability dye (1:1,000; 423,106, BioLegend) and incubated for 30 min at 37 °C/5% CO₂ in the dark. Following incubation, the samples were immediately placed on ice and analyzed by flow cytometry.

### MPO-DNA ELISA

MPO-DNA complexes (i.e. NETs) were quantified as previously described (Zuo et al. 2020), with modifications (Pastorek et al. 2023). High-binding 96-well plates (82.1581.210, Sarstedt) were coated overnight at 4 °C with anti-MPO antibody (0.5 µg/ml; 0400–0002, Bio-Rad) in 100 mM carbonate-bicarbonate buffer. Plates were washed with PBS + 0.05% Tween 20 (AppliChem, Darmstadt, Germany) and blocked with 4% BSA + 0.05% Tween 20 in PBS for 90 min at RT. Samples diluted 100 × in blocking buffer were incubated for 90 min at RT, followed by anti-DNA-POD detection antibody (1:100; 11,774,425,001, Roche) for 90 min at RT. After five washes, plates were developed with TMB substrate (Life Technologies, Carlsbad, CA, USA) and stopped with 0.16 M H₂SO₄ (R&D Systems); absorbance was read at 450 nm (Synergy H4, BioTek).

### TLR9-mediated NF-κB reporter assay

HEK-Dual™ hTLR9 (NF-κB/IL-8 reporter) and TLR9-null control cells (hkd-htlr9ni, hkd-nfis, InvivoGen, San Diego, CA, USA) were maintained in DMEM (Gibco) + 10% FBS at 37 °C/5% CO₂ under selective antibiotics per manufacturer's instructions. Cells (1 × 10^5^/well) were stimulated for 24 h with intact MITO or mtDNA derived from ~ 1 × 10⁸ mitochondria equivalents. ODN 1826 (50 nM) and TRIDAP (1 µg/ml; tlrl-1826c-1, tlrl-tdap, InvivoGen) served as positive controls. NF-κB-driven reporter activity was measured by absorbance at 620 nm (Synergy H4, BioTek).

### mtDNA stability assay

Intact MITO, Proteinase K-treated MITO, or isolated mtDNA (~ 1 × 10⁷ MITO equivalents in 15 µl PBS) were mixed with 100 µl healthy donor plasma and 100 µl reaction buffer (0.1 mM CaCl₂, 0.3 mM MgCl₂, 10 mM Tris–HCl, pH 7.4) and incubated at 37 °C for 30 min. DNA was extracted using the QIAamp DNA Blood Mini Kit (51,106, Qiagen; elution volume 50 µl) and quantified by qPCR as described in "Mitochondria isolation and processing" section. For kinetic analysis, SYTOX™ Green (400 nM) was added to the reaction buffer and fluorescence was recorded every 5 min for 120 min at 37 °C (Synergy H4, BioTek).

## Animal experiments

### Animals and housing

Male C57BL/6 J mice (6 months; Velaz, Prague, Czech Republic) were housed under a 12 h light/dark cycle at 24 ± 2 °C and 55 ± 10% humidity with ad libitum access to standard chow and water.

### Surgery-related traumatic injury model

Mice were anesthetized by i.p. injection of ketamine (100 mg/kg) and xylazine (10 mg/kg). A midline laparotomy was performed with sterile scissors: a 1 cm incision was made through skin and abdominal wall, and sterile tweezers were introduced between intestinal loops and moved in slow circular motions for 10 s to simulate tissue trauma, followed by layered suture closure. To model circulating mitochondrial release, a separate group received ~ 4 × 10⁸ MITO in 100 µl sterile PBS i.p. Mice were euthanized at 0, 1, and 4 h post-procedure by terminal retro-orbital blood collection into EDTA tubes (Microvette, Sarstedt) followed by cervical dislocation. Peritoneal lavage fluid (PLF) was collected immediately.

### Double-hit model of sterile injury followed by pneumonia

Following the adapted double-hit animal model protocol by Itagaki et al., mice were restrained and received FPRA14 (16.18 µg/kg, i.v.; MW calculated to correspond to the 500 nM dose used in vitro) or vehicle (PBS) at hour − 1, followed by ~ 4 × 10⁸ MITO in 100 µl PBS i.p. at hour 0. At hour 3, mice were anesthetized with ketamine (50 mg/kg) and xylazine (5 mg/kg) i.p. and inoculated intratracheally with *S. aureus* (50 µl of OD₆₀₀ = 0.4 suspension in PBS) (Itagaki et al. 2020). At hour 19, mice were anesthetized by inhalation (3% isoflurane/97% O₂) and terminally bled via the retro-orbital sinus, followed by PLF collection and cervical dislocation. For bronchoalveolar lavage (BALF), the right lung lobe was washed with 1 ml PBS via a 22-G catheter. The left lung lobe was homogenized as a 10% (w/v) suspension in sterile PBS using stainless-steel beads and a TissueLyser II (Qiagen). For bacterial clearance quantification, lung homogenate was serially diluted in PBS, spot-plated (10 µl) on LB-agar, incubated overnight at 37 °C, and CFU counted manually.

### Flow cytometry

PLF and BALF were centrifuged at 400 × g, 10 min, 4 °C; pellets were resuspended in FC buffer (phenol red-free RPMI 1640 + 1% FBS) and stained for 20 min at RT in the dark with: anti-mouse Ly-6G Alexa Fluor® 647 (2.5 µg/ml; 127,610, BioLegend), anti-mouse CD11b BV605 (2 µg/ml; 101,257, BioLegend), anti-histone H3 (1:2,000; ab219407, Abcam, Cambrige, United Kingdom), donkey anti-rabbit IgG BV510 (0.4 µg/ml; 406,419, BioLegend), and SYTOX™ Green (50 nM). Ly-6G⁺ CD11b⁺ cells were classified as neutrophils; NETotic neutrophils were defined as histone H3⁺ SYTOX™ Green⁺. Lavage supernatants were diluted 1:100 in PBS and stained with MitoTracker Green FM (100 nM; M7514, Invitrogen) and TO-PRO-3 iodide (500 nM; T3605, Invitrogen) for 20 min at RT to detect extracellular mitochondrial particles with the flow cytometer setup as previously described (Lichá et al. 2023). Briefly, flow cytometer was pre-calibrated using calibration beads of sizes of 100 nm, 200 nm, 500 nm and 1000 nm. The gating strategy was used as follows: all events were plotted using a V-SSC-H channel on an *x*-axis and an FITC-A channel on a *y*-axis. Signal noise was excluded based on the Flow Cytometry Sub-Micron Sizer Reference Kit and Green Fluorescent beads (F13839, Thermo Fisher) by the selection of events with fluorescence ≥ 100 nm FITC-labeled beads. Particular size gates were then set up according to 100 nm, 200 nm and 500 nm bead populations and further verified by creating a calibration curve from the V-SSC-H median of the corresponding population. Human plasma samples were processed identically after centrifugation at 1,600 × g, 10 min, 4 °C. All samples were acquired on a DxFlex cytometer (Beckman Coulter, Brea, CA) and analyzed with FCS Express 7 (De Novo Software, Pasadena, CA, USA).

### Cytospin and Wright-Giemsa staining

PLF cell pellets were resuspended in 50 µl PBS and cytocentrifuged at 1,800 rpm for 7 min (Centric 250, Domel, Železniki, Slovenia) onto glass slides. Slides were air-dried, fixed in 4% paraformaldehyde for 2 min, stained using a Wright-Giemsa three-step kit (Richard-Allan Scientific™, Kalamazoo, MI, USA), and examined on a Leica DM2500 microscope (Leica, Wetzlar, Germany).

### Cytokine profiling

Pro- and anti-inflammatory cytokines in PLF and BALF were quantified using the LEGENDplex™ Mouse Inflammation Panel (740,446, BioLegend) per the manufacturer's instructions. Data were log-transformed prior to statistical analysis. Values below the limit of detection (LOD) were treated as left-censored at LOD and were normalized as LOD/√2 prior to analysis.

### Oxidative damage analysis

Supernatant from BALF was analyzed for markers of oxidative stress. Measurement of advanced oxidation protein products (AOPP) was quantified using a method adapted from Witko-Sarsat, et al., and thiobarbituric acid reactive substances (TBARS) were measured according to the protocol described Draper et al. (Witko-Sarsat et al. 1998; Draper et al. 1993). Briefly, AOPP was measured after the addition of glacial acetic acid at 340 nm. For TBARS, BALF supernatants were boiled at 95 °C for 45 min with thiobarbituric acid and glacial acetic acid. Following n-butanol extraction, the fluorescence of the organic phase was measured at an excitation wavelength of 515 nm and an emission wavelength of 553 nm. For measurement of oxidative damage, DNA was isolated from BALF supernatant using QIAamp DNA Blood Mini Kit for subsequent analysis of 8-hydroxy-2'-deoxyguanosine (8-OHdG) according to manufacturer's instructions (K059-H5, DNA Damage Enzyme Immunoassay Kit, Arbor Assays, Ann Arbor, MI, USA).

## Statistical and Chou-Talay combination index analysis

All statistical analyses were performed in GraphPad Prism v10.0 (GraphPad Software, La Jolla, CA, USA). Data are presented as individual values; each dot represents a single observation. Group comparisons used Kruskal–Wallis test with Dunn's post hoc test, or two-way ANOVA with Šidák’s multiple comparison test, as appropriate, unless otherwise stated. Drug interaction synergy/antagonism was analyzed using the Chou-Talalay combination index method implemented in CalcuSyn v2.0 (Biosoft, Cambridge, UK). A p-value < 0.05 was considered statistically significant.

## Results

### Mitochondria released during surgical trauma drive NET formation in vivo

**Fig. 1:**
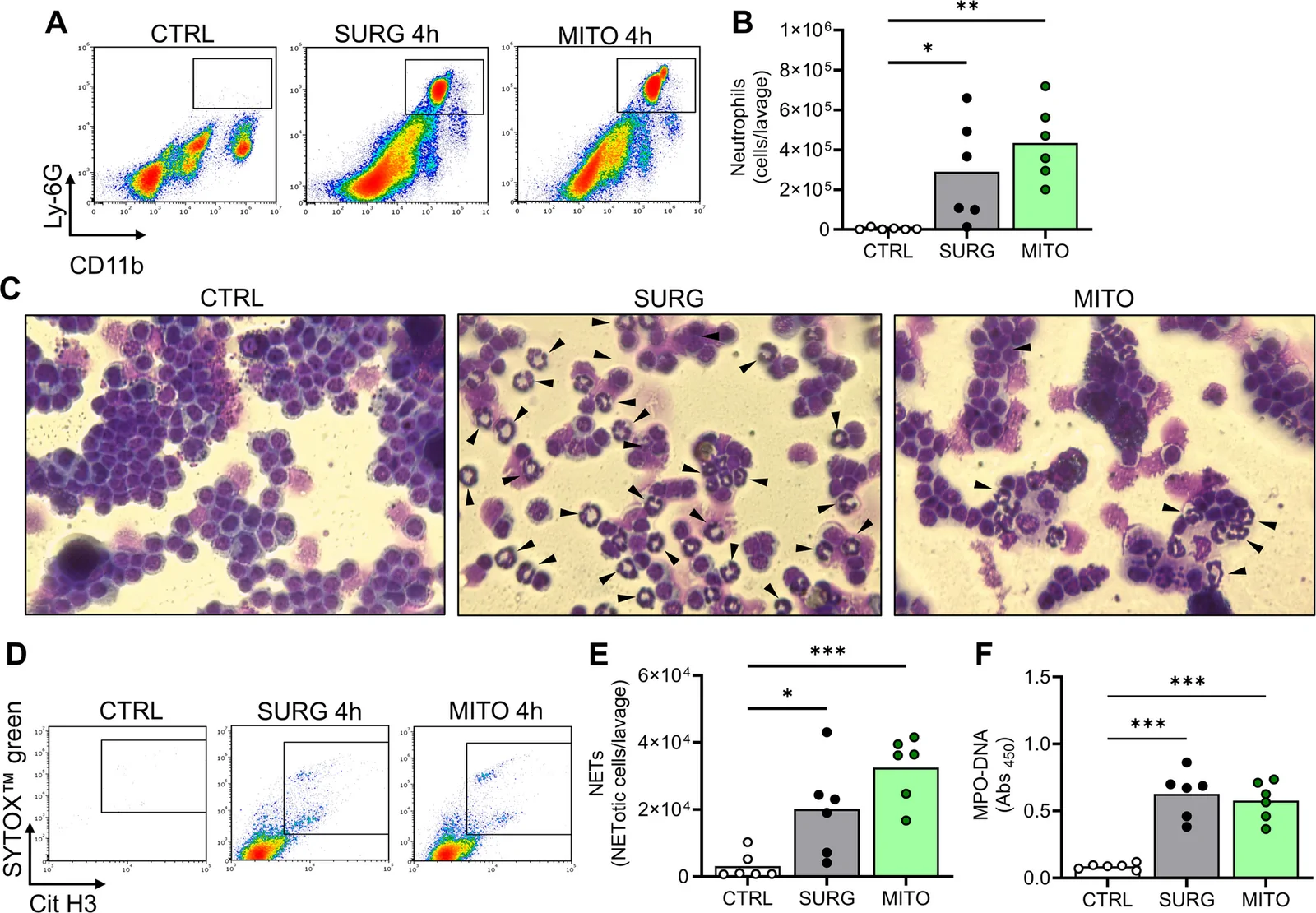
Mitochondria released during surgical trauma attract neutrophils and drive NET formation in vivo. Mice were subjected to laparotomy (SURG) or intraperitoneal injection of isolated mitochondria (MITO; 4 × 10^8^ particles) and sacrificed at the indicated time points. Peritoneal lavage fluid (PLF) was collected and analyzed. **A** Representative flow cytometry plots and **B** quantification of Ly-6G⁺ CD11b^+^ neutrophils in PLF at 4 h post-procedure. **C** Representative cytospin preparations of PLF cells 4 h post-procedure. Black arrowheads indicate polymorphonuclear cells. Scale bar = 50 μm. **D** Representative flow cytometry plots and **E** quantification of NETotic neutrophils identified as Ly-6G⁺ CD11b^+^ events positive for SYTOX™ Green and citrullinated histone H3 (Cit H3). **F** MPO-DNA complex levels in PLF at 4 h post-procedure as a biochemical marker of NET formation. Data are presented as the mean with individual datapoints; *n* = 6 per group. *p < 0.05, **p < 0.01, ***p < 0.001 vs. CTRL; Kruskal–Wallis test with Dunn's post hoc test

It is well established that mtDAMPs activate neutrophil responses, including NET formation, being implicated in pathological inflammation associated with sterile trauma (Hauser and Otterbein 2018). To first confirm this biology in vivo, we subjected mice to laparotomy (surgery-related trauma, SURG) or intraperitoneal injection of isolated mitochondria (MITO) and compared the inflammatory response in peritoneal lavage fluid (PLF). Mitochondrial particles were detected in PLF as early as one hour post-surgery (5.5 × 10^7^ particles/lavage, *p* < 0.05), returning to baseline by 4 h. Exogenously administered mitochondria showed analogous kinetics, with peak concentrations immediately after injection (7.6 × 10^7^ particles/lavage, *p* < 0.01) rapidly declining thereafter, consistent with mitochondrial clearance post-surgery (S2 Fig). Both models induced robust neutrophil recruitment by 4 h post-procedure (SURG: 4.5 × 10^3^ vs. 2.9 × 10^5^ cells/lavage *p* < 0.05; MITO: 4.3 × 10^5^ cells/lavage *p* < 0.01; Fig. 1A, B), confirmed morphologically in cytospin preparations (Fig. 1C). Critically, both SURG and MITO triggered NET formation, evidenced by significantly elevated numbers of NETotic neutrophils in lavage (SURG: 3107 → 20,143 NETotic cells/lavage, *p* < 0.05; MITO: 3107 → 32,510 NETotic cells/lavage, *p* < 0.001) (Fig. 1D, E, S3 Fig). NETs formation in both models was confirmed by individual analysis of PLF concentration of MPO-DNA complexes (SURG: AU 0.087 → 0.627, *p* < 0.001; MITO: 0.14 → 0.577, *p* < 0.001; Fig. 1F). These data confirm that extracellular mitochondria are sufficient to drive sterile neutrophil activation and NET formation in vivo. Isolated mitochondria were therefore used as a defined stimulus throughout this study to dissect the mechanisms of mtDAMP-driven NETosis and to evaluate whether this response can be selectively attenuated without compromising antimicrobial neutrophil function.

## mtDAMP-driven NETosis depends on mitochondrial integrity and engages multiple NETosis pathways

**Fig. 2:**
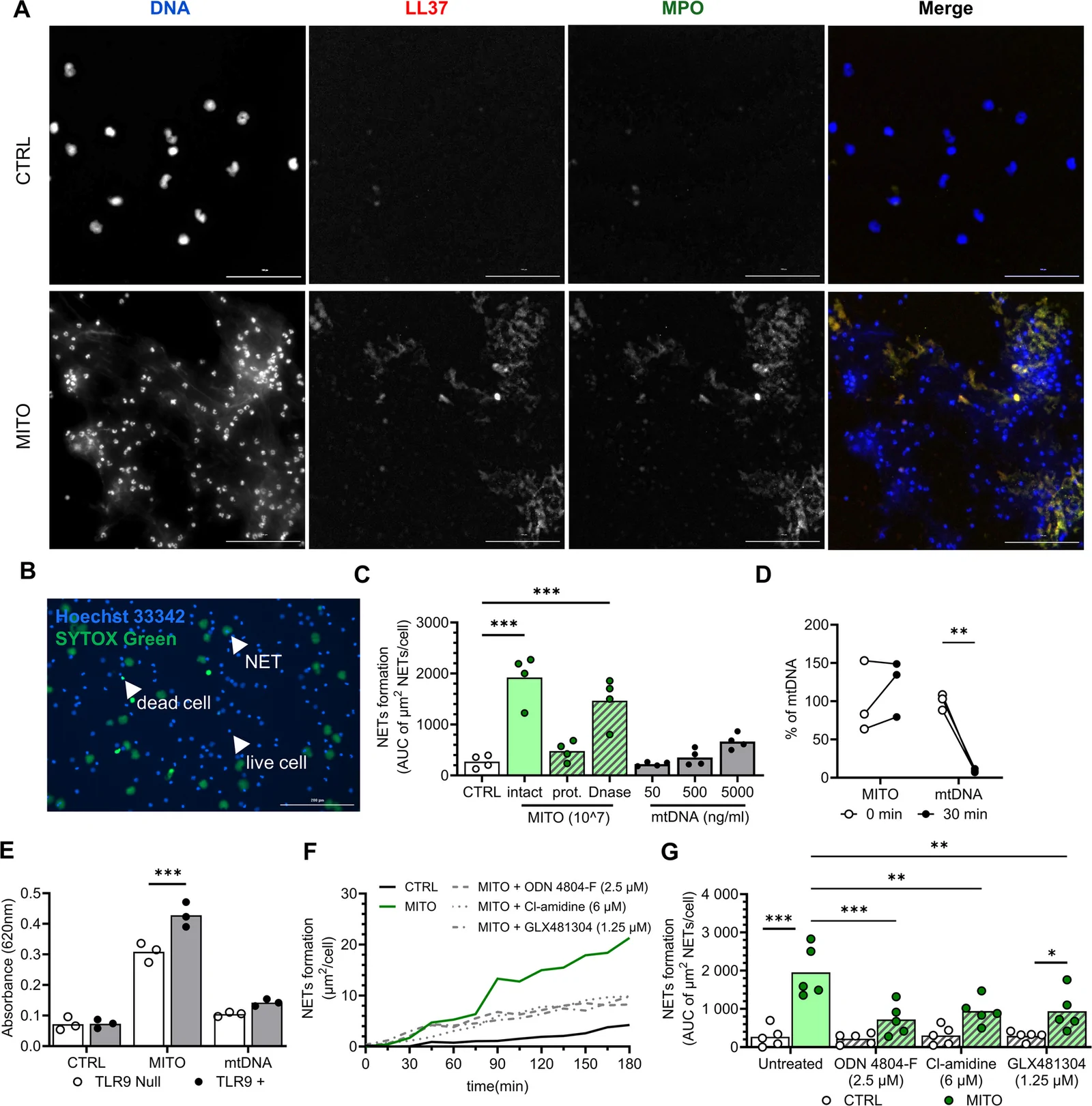
Intact mitochondria drive NET formation more potently than individual mtDAMPs, engaging multiple signaling pathways. Isolated human neutrophils were co-incubated with intact or processed mitochondria (MITO; 1 × 10⁷ particles) or isolated mitochondrial components for 3 h at 37 °C. PMA (100 nM) served as positive control. **A** Representative immunofluorescence images of CTRL and MITO-stimulated neutrophils stained for DNA (Hoechst 33,342, blue), LL37 (red), and MPO (green). Merged images are shown in the rightmost panels. Scale bar = 50 μm. **B** Representative live-cell imaging frame (Hoechst 33,342/SYTOX™ Green) with annotated NET, dead cell, and live cell populations. **C** Total NET formation quantified as AUC (of µm^2^ NETs/cell) in neutrophils co-incubated 180 min with intact MITO, proteinase K-treated MITO (prot.), DNase-treated MITO, or isolated mtDNA at indicated concentrations. **D** Mitochondrial genome copy number (% of input) measured by qPCR in donor plasma spiked with MITO or isolated mtDNA, before (0 min) and after (30 min) incubation. **E** NF-κB reporter activation (absorbance at 620 nm) in TLR9-expressing (TLR9 +) and TLR9-null reporter cells following stimulation with CTRL, MITO, or mtDNA. **F** NET formation kinetics (μm^2^/cell) over 180 min in neutrophils pre-treated with TLR9 antagonist (ODN 4804-F), PAD4 inhibitor (Cl-amidine), or NOX2 inhibitor (GLX481304) prior to MITO stimulation. **G** Total NET formation quantified as AUC (μm^2^ NETs/cell) for conditions shown in (F). Data are presented as the mean with individual datapoints; n = 3–6 per group. *p < 0.05, **p < 0.01, ***p < 0.001 vs. CTRL; **C** Kruskal–Wallis test with Dunn's post hoc test. *p < 0.05, **p < 0.01, ***p < 0.001; **D-G** two-way ANOVA with Šidák’s multiple comparison test

To determine which mitochondrial component dominantly drives NET formation, we co-incubated isolated human neutrophils (2 × 10^4^/well) with intact or enzymatically processed mitochondria (1 × 10⁷ particles/well) for 3 h. NET formation was quantified by live-cell imaging and confirmed by fluorescent microscopy (Fig. 2A, B).

Intact MITO induced NET formation (271 AU vs. 1922 AU; *p* < 0.001), as did DNase-treated MITO (271 AU vs. 1466 AU; *p* < 0.001). In contrast, proteinase K-treated MITO failed to induce NET formation above baseline (271 AU vs. 479 AU; p > 0.05) (Fig. 2C). Isolated mtDNA alone (50–5000 ng/ml), was similarly insufficient even at the highest concentration of 5000 ng/ml (271 AU vs. 666 AU, p > 0.05) (Fig. 2C). To assess whether this reflected intrinsic instability of isolated mtDNA, we spiked donor plasma with either intact MITO or isolated mtDNA and quantified mitochondrial genome copy number by qPCR after 30 min. mtDNA copy number fell to 9% of input when administered alone (*p* < 0.01), but remained stable in intact MITO (Fig. 2D). Complementary analysis of fluorescently labelled mtDNA also showed that while mtDNA within intact mitochondria remained stable over time, both proteinase K-treated and isolated mtDNA underwent a comparable decline (S4 Fig). Individual lipid and protein components of mitochondria, cardiolipin and cytochrome C, induced very little NET formation (271 AU vs. 582 AU and 659 AU) (S5 Fig).

To further characterize how isolated or organelle-bound mtDNA affects TLR9 activation, we assessed the immunogenicity of intact MITO versus isolated mtDNA using a TLR9-mediated NF-κB reporter system. Intact MITO activated both TLR9-expressing and TLR9-null reporter cells (0.073 AU vs. 0.428 AU and 0.072 AU vs 0.308 AU, respectively, both *p* < 0.001), reflecting TLR9-independent triggering components present within the intact organelle. Critically, the differential in NF-κB activation between TLR9-expressing and TLR9-null cells was markedly greater for intact MITO than for isolated mtDNA (0.428 AU vs. 0.142 AU, *p* < 0.001), which is consistent with the previously observed higher stability of protected mtDNA (Fig. 2E, S4 Fig). To further dissect the signaling mechanisms underlying MITO-driven NET formation we tested TLR9 inhibition with inhibitory oligo ODN 4804-F (S4 Fig), PAD4 inhibition with Cl-amidine, and NOX2 inhibition with GLX481304. All three inhibitors significantly reduced NET formation, with divergence from untreated neutrophils apparent beyond 75 min of treatment. Specifically, TLR9 inhibition with ODN 4804-F reduced NET formation from 1829 to 720 AU (*p* < 0.01); PAD4 inhibition with Cl-amidine reduced it to 940 AU (*p* < 0.01); and NOX2 inhibition with GLX481304 reduced it to 938 AU (*p* < 0.01) (Fig. 2F, G).

## FPR1 saturation attenuates MITO-driven NET formation in vitro and in trauma plasma

**Fig. 3:**
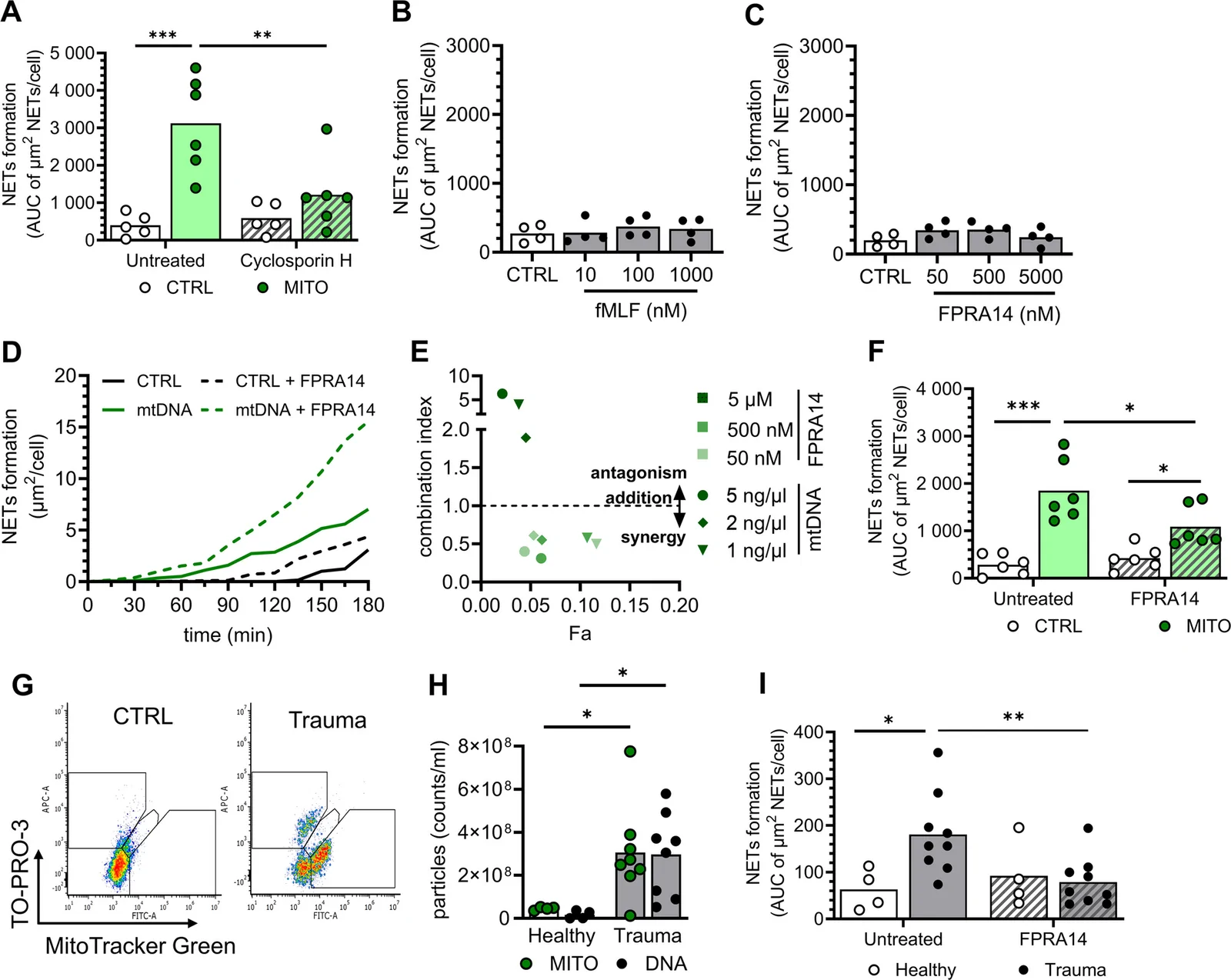
FPR1 regulates MITO-driven NETosis and its saturation attenuates NET formation induced by trauma plasma. Isolated human neutrophils were stimulated with MITO (1 × 10⁷ particles), FPR1 agonists, or plasma from trauma patients for 180 min at 37 °C. PMA (100 nM) served as positive control unless otherwise stated. **A** Total NET formation (AUC of µm^2^ NETs/cell) in neutrophils pre-treated with FPR1 antagonist Cyclosporin H prior to MITO stimulation. **B** Total NET formation (AUC of µm^2^ NETs/cell) following stimulation with fMLF at indicated concentrations (10–1000 nM). **C** Total NET formation (AUC of µm^2^ NETs/cell) following stimulation with FPRA14 at indicated concentrations (50–5000 nM). **D** NET formation kinetics (µm^2^/cell) over 180 min in neutrophils co-incubated with mtDNA (2 ng/µl), FPRA14 (500 nM), or their combination. **E** Chou-Talalay combination index (CI) analysis and Fraction affected (Fa) calculation of mtDNA and FPRA14 interactions across a concentration matrix. CI < 1 indicates synergy; CI > 1 indicates antagonism; CI = 1 indicates additivity. **F** Total NET formation (AUC of µm^2^ NETs/cell) in neutrophils pre-treated with FPRA14 (500 nM) prior to MITO stimulation. **G** Representative flow cytometry plots of plasma from healthy donors and trauma patients, stained with MitoTracker Green and TO-PRO-3 to identify mitochondrial and DNA-positive particles respectively. **H** Quantification of MitoTracker-positive (MITO) and TO-PRO-3-positive (DNA) particle counts in plasma from healthy donors and trauma patients. **I** Total NET formation (AUC of µm^2^ NETs/cell) in healthy donor neutrophils incubated with plasma from healthy donors or trauma patients, with or without FPRA14 pre-treatment. Data are presented as the mean with individual datapoints; n = 4–9 per group. *p < 0.05, **p < 0.01, ***p < 0.001 vs. CTRL; **B**, **C** Kruskal–Wallis test with Dunn's post hoc test. *p < 0.05, **p < 0.01, ***p < 0.001; **A**, **F-I** two-way ANOVA with Šidák’s multiple comparison test

Having established the importance of mitochondrial proteins in MITO-driven NET formation, we next considered whether they may serve additional roles beyond mtDNA stabilization. Given that mitochondria are a rich source of N-formylated peptides recognized by FPR1, we asked whether FPR1 engagement contributes to NET formation induced by intact mitochondria. To address this, we pre-treated neutrophils with Cyclosporin H, a selective FPR1 antagonist, 30 min prior to MITO stimulation. FPR1 inhibition significantly reduced NET formation compared to untreated MITO-stimulated neutrophils (3121 AU vs. 1216 AU, *p* < 0.01) (Fig. 3A). However, stimulation with individual selective FPR1 agonists such as the established bacterial fMLF (10–1000 nM) or the synthetic agonist FPRA14 (50–5000 nM) failed to induce NET formation on their own (*p* > 0.05) (Fig. 3B, C).

Since no individual stimulus was sufficient to replicate the intensity of NET formation induced by intact mitochondria, we asked whether simultaneous engagement of both FPR1 (via FPRA14 or bacterial fMLF) and TLR9 (via mtDNA) could reconstitute this response. While either stimulus alone produced only a modest increase in NET release, their concurrent combination yielded either robust synergistic induction or conversely attenuation of NET formation (Fig. 3D). Chou-Talalay combination index analysis revealed concentration-dependent interaction: the highest concentration of FPRA14 tested (5 µM), when combined with all mtDNA concentrations, produced antagonism (CI 1.89–6.26; Fa < 0.05). In contrast, moderate (500 nM) and low (50 nM) concentrations of FPRA14 acted synergistically with mtDNA across all tested concentrations (CI < 0.61; Fa > 0.04), with the strongest synergism observed at 50 nM FPRA14 combined with 1 ng/µl mtDNA (CI = 0.31; Fa = 0.12; Fig. 3E). An analogous pattern was observed when fMLF (10–1000 nM) was substituted for FPRA14 (S6 Fig).

These findings identified FPR1 agonist concentration as the primary determinant of NET formation outcome during concurrent FPR1 and TLR9 engagement, with high concentrations suppressing rather than enhancing the response. We thus hypothesized that excessive FPR1 stimulation drives receptor desensitization, ultimately blunting its co-stimulatory contribution to TLR9-initiated NETosis. Extending agonist exposure over time produced a similar effect: 30-min pre-treatment of neutrophils with 500 nM FPRA14 prior to mitochondria stimulation significantly reduced total NET formation compared to controls (1850 AU vs. 1089 AU, *p* < 0.05) (Fig. 3F).

To evaluate the translational relevance of these findings, we have next quantified circulating mtDAMPs in trauma patient plasma, analyzed its capacity to induce NET formation, and evaluated whether FPRA14 pre-treatment could attenuate this response. Flow cytometric analysis revealed significantly elevated concentrations of both MitoTracker-positive mitochondrial particles (3.1 × 10⁸ vs. 4.6 × 10⁷ particles/ml, *p* < 0.05) and TO-PRO-3-positive DNA-containing particles (3 × 10⁸ vs 1.7 × 10⁷ particles/ml, *p* < 0.05) in trauma patient plasma compared to healthy donor plasma (Fig. 3G, H, S2 Fig). Trauma patient plasma induced significantly greater NET formation in healthy donor neutrophils relative to healthy donor plasma (63 AU vs. 181 AU, *p* < 0.01). Notably, 30-min pre-treatment with 500 nM FPRA14 attenuated this response (181 AU vs. 79 AU, *p* < 0.01) (Fig. 3I).

## NET formation induced by mitochondria is selectively targeted by FPRA14 pre-treatment

**Fig. 4:**
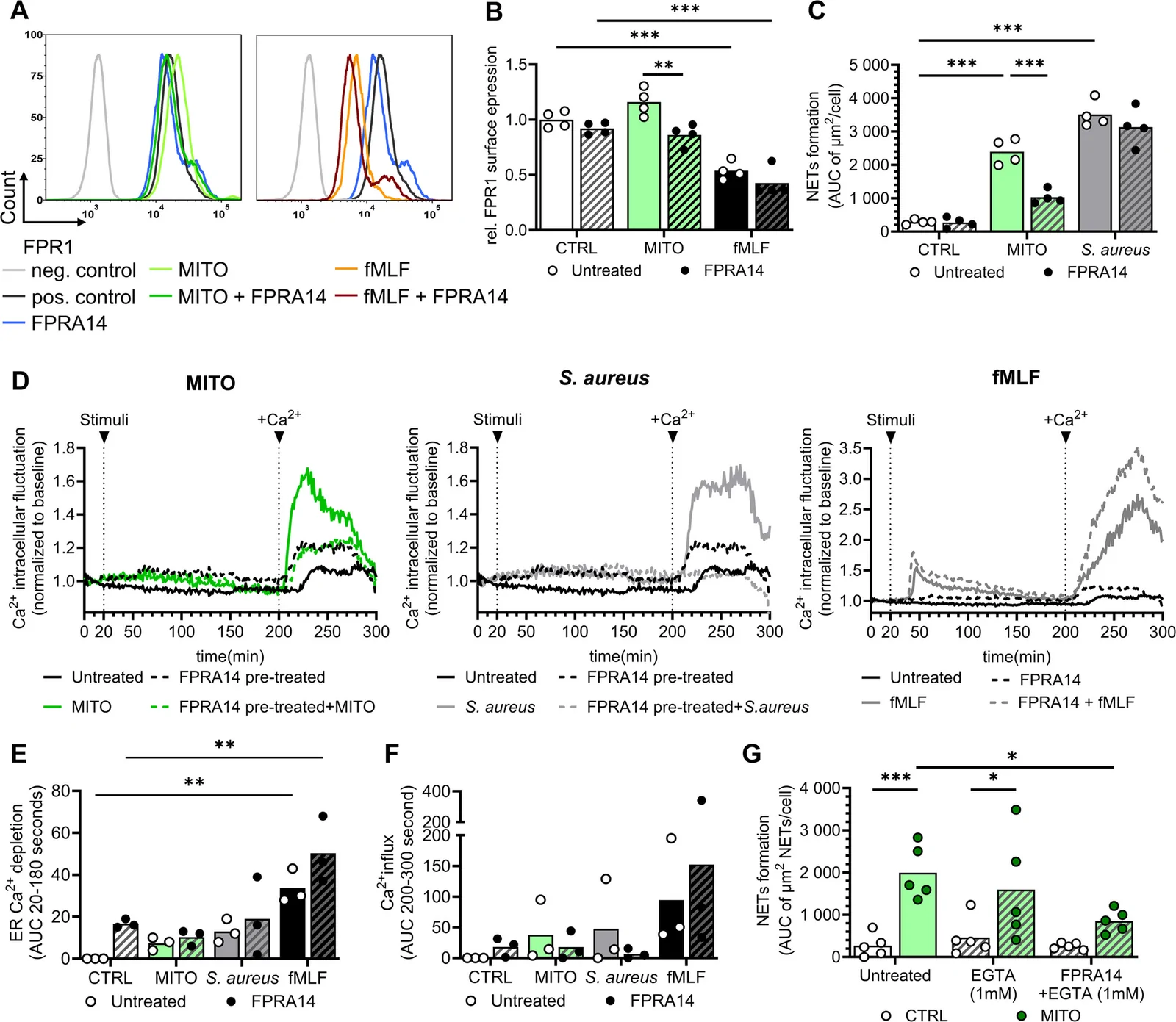
Mitochondria FPR1 engagement drives NETosis and is selective by FPRA14 pre-treatment independently of Ca^2^⁺ signaling. Isolated human neutrophils were pre-treated with FPRA14 (500 nM) or left untreated prior to stimulation with PMA (100 nM), fMLF (100 nM), MITO (1 × 10⁷ particles), or *S. aureus* (1 × 10⁷ particles) for the indicated durations at 37 °C. **A** Representative flow cytometry histograms showing surface FPR1 expression (FITC-A) in neutrophils co-incubated with fMLF or MITO, with or without FPRA14 pre-treatment. Left panel shows conditions with partial internalization (MITO, MITO + FPRA14); right panel shows conditions with stronger receptor internalization (fMLF, fMLF + FPRA14, FPRA14 alone). **B** Quantification of relative surface FPR1 expression normalized to unstimulated control neutrophils, across fMLF and MITO conditions with or without FPRA14 pre-treatment. **C** Total NET formation (AUC of µm^2^ NETs/cell) following co-incubation with PMA, MITO, or *S. aureus* in untreated or FPRA14-pre-treated neutrophils. **D** Intracellular Ca^2^⁺ kinetics (normalized to baseline) in untreated and FPRA14-pre-treated neutrophils following stimulation with MITO (left), *S. aureus* (middle), or *S.* fMLF (right). Dashed vertical lines indicate the time of stimulus addition and subsequent extracellular Ca^2^⁺ supplementation. **E** Quantification of endoplasmic reticulum (ER) Ca^2^⁺ depletion expressed as AUC over 20–180 s following stimulus addition, across all conditions. **F** Quantification of extracellular Ca^2^⁺ influx expressed as AUC over 200–300 s following extracellular Ca^2^⁺ addition, across all conditions. **G** Total NET formation (AUC of µm^2^ NETs/cell) in MITO-stimulated neutrophils in the presence or absence of EGTA (1 mM) and/or FPRA14 pre-treatment. Data are presented as the mean with individual datapoints; n = 3–5 per group. *p < 0.05, **p < 0.01, ***p < 0.001; two-way ANOVA with Šidák’s multiple comparison test

To confirm that intact mitochondria interact directly with FPR1, we quantified surface FPR1 expression by flow cytometry following 20-min co-incubation with fMLF or MITO in untreated neutrophils (Fig. 4A). fMLF induced receptor internalization more potently than MITO, where we observed an increase in surface expression (→ 53.76% of baseline; *p* < 0.001, vs. → 115.95% of baseline; *p* < 0.05, respectively) (Fig. 4B). Building on this, we next explored whether FPRA14 could selectively prevent MITO from engaging the receptor. Since MITO triggers only partial FPR1 internalization, we reasoned that pre-occupying the receptor with FPRA14 may be sufficient to block MITO-FPR1 engagement, while stronger agonists would be expected to overcome this pre-occupancy. FPRA14 alone has not significantly reduced surface FPR1 compared to the baseline (p > 0.05). Notably, while FPRA14 pre-treatment did not significantly affect surface FPR1 in neutrophils co-incubated with fMLF (53.76 → 42.62%), it did so in MITO-stimulated neutrophils (115.954% → 86.24%; *p* < 0.01; Fig. 4B). We therefore next asked whether this selective receptor-level blockade could translate into functional suppression of MITO-driven NET formation without broadly compromising NET formation against pathogens.

To assess this, neutrophils were pre-treated with 500 nM FPRA14 and subsequently co-incubated with PMA (100 nM), MITO, or *S. aureus* (both 1 × 10⁷ particles/bacteria per reaction). We observed that FPRA14 pre-treatment reduced NET formation in MITO-stimulated neutrophils (2399 AU vs. 1030 AU, *p* < 0.001), while the response to *S. aureus* was not significantly affected (3512 AU vs 3136 AU, p > 0.05) (Fig. 4C). We verified the specificity of FPR1 using parallel non-specific FPR agonist FPRA43 and inhibitor Cyclosporin H. All three substances reduced NETs formation to MITO, highlighting FPRA14 highest efficiency (mitochondria 2290 AU vs. FPRA14 983 AU, *p* < 0.001; vs. FPR43 1522 AU, *p* < 0.01; vs. Cyclosporin H 1450 AU, *p* < 0.01). Similarly, general pre-treatment modulation of FPR did not affect NET formation in response to *E. coli* (S7 Fig). To investigate the downstream signaling mechanism, we measured intracellular Ca^2^⁺ kinetics in untreated and FPRA14-pre-treated neutrophils following stimulation with fMLF, MITO, or *S. aureus*, as FPR1 activation canonically triggers Ca^2^⁺ mobilization through endoplasmic reticulum (ER) store release followed by extracellular influx (Fig. 4D). ER Ca^2^⁺ depletion was significantly elevated only in both untreated and FPRA14 pre-treated fMLF-stimulated neutrophils (both 33.7 AUC increase, *p* < 0.01); no significant differences were observed for MITO or *S. aureus* (both p > 0.05) (Fig. 4E). Extracellular Ca^2^⁺ influx did not differ significantly across stimuli, and FPRA14 pre-treatment showed only modest, non-significant trends in all conditions (p > 0.05 for all) (Fig. 4F). To further verify if extracellular Ca^2^⁺ availability does not affect MITO-driven NETosis, we chelated extracellular Ca^2^⁺ with EGTA (1 mM). EGTA alone did not significantly reduce NET formation (p > 0.05), and only after the addition of FPRA14 pre-treatment have we observed reduction in total NETosis (1996 AU vs. 853 AU, *p* < 0.05) (Fig. 4G).

## Distinct phosphorylation signatures underlie MITO- and *S. aureus*-driven NETosis, with p38α and STAT6 as selective modulators

**Fig. 5:**
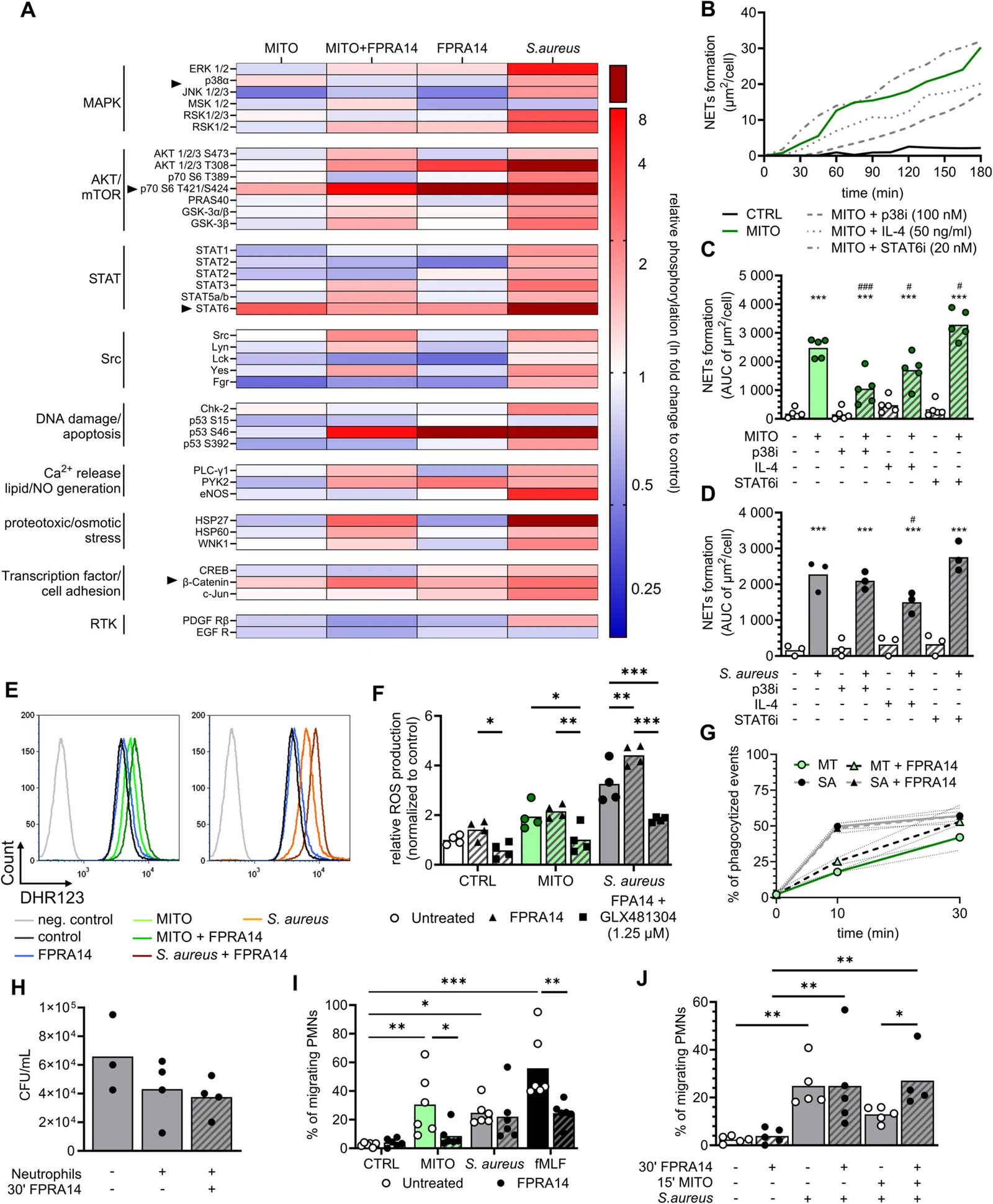
Distinct phosphorylation underlies MITO- and S. aureus-driven NETosis and FPR1 desensitization selectively modulates neutrophil functions. A Heatmap of relative phosphorylation levels (log fold change to untreated control) of key signaling kinases in neutrophils stimulated with MITO, MITO + FPRA14, FPRA14 alone, or S. aureus. Arrows indicate kinases with selective upregulation in MITO-stimulated neutrophils (p38α, p70 S6K T421/S424, STAT6, β-Catenin) that were attenuated by FPRA14 pre-treatment. Color scale ranges from 0.25 (blue, decreased) to 8 (dark red, increased). B Kinetics of NET formation (µm2/cell) over 180 min in MITO-stimulated neutrophils pre-treated with vehicle control, IL-4 (50 ng/ml), p38i (100 nM), or STAT6i (20 nM). C Total NET formation (AUC of µm2/cell) across all conditions, including combinations of FPRA14 with p38i, STAT6i, or IL-4 towards MITO and D S. aureus. E Bacterial load (CFU/mLl) following co-incubation of S. aureus with untreated or FPRA14-pre-treated neutrophils. F Percentage of phagocytosing neutrophils after 10 and 30 min of co-incubation with MITO (MT) or S. aureus (SA), in untreated or FPRA14-pre-treated neutrophils. G Percentage of migrating neutrophils (PMNs) in Transwell chemotaxis assays toward fMLF, MITO, or S. aureus, in untreated or FPRA14-pre-treated neutrophils. H Percentage of migrating PMNs toward S. aureus following sequential treatment: 30-min FPRA14 pre-treatment, 15-min MITO co-incubation, and subsequent S. aureus chemotaxis assay. I Representative flow cytometry histograms showing intracellular ROS content of neutrophils co-incubated with MITO or S. aureus, with or without FPRA14 pre-treatment. Left panel represents control samples and MITO-treated neutrophils, right panel showing control samples and S. aureus-treated neutrophils. J Relative intracellular ROS production in neutrophils normalized to control untreated neutrophils after 30 min of co-incubation with MITO or S. aureus. Data are presented as the mean with individual datapoints; n = 3–6 per group. *p < 0.05, **p < 0.01, ***p < 0.001; ###p < 0.001, ##p < 0.01 (FPRA14-pre-treated vs. untreated within condition); C-E Kruskal–Wallis test with Dunn's post hoc test; F–H, J two-way ANOVA with Šidák’s multiple comparison test

Since Ca^2^⁺ mobilization appeared dispensable for MITO-driven NET formation, we investigated the involvement of other signaling molecules downstream of FPR1. We therefore profiled the phosphorylation state of key kinases in MITO- and MITO + FPRA14-stimulated neutrophils. In parallel, we included *S. aureus*-stimulated neutrophils as a reference condition for pathogen-driven neutrophil activation. *S. aureus*-stimulated neutrophils displayed broad upregulation of phosphorylation across nearly all assayed kinases (Fig. 5A). In contrast, MITO-stimulated neutrophils showed a more selective phosphorylation signature, with notable increases restricted to p38α (21% increase), p70 S6K (T421/S424) (58% increase), STAT6 (143% increase), and β-Catenin (31% increase) (indicated by arrows, Fig. 5A). Pre-treatment with FPRA14 attenuated phosphorylation of p38α and STAT6 (by 37% and 71%, respectively) in MITO-stimulated neutrophils (Fig. 5A).

We therefore focused on p38α and STAT6 as candidate mediators of MITO-driven NET formation. To modulate these pathways, neutrophils were pre-treated with a p38α inhibitor (BIRB 796-p38i; 100 nM), a STAT6 inhibitor (AS1517499-STAT6i; 20 nM), or IL-4 (50 ng/ml) to pre-activate the STAT6 pathway, prior to MITO stimulation. Inhibition of STAT6 accelerated the onset of NET formation compared to untreated neutrophils, while pre-activation of STAT6 with IL-4 and inhibition of p38α both delayed NET formation (Fig. 5B). Quantification confirmed that p38α inhibition and IL-4 pre-treatment significantly reduced overall NET formation to MITO (2475 AU vs. 1050 AU, *p* < 0.001; 2542 AU vs. 1702 AU, *p* < 0.05, respectively). Conversely, increase of NET formation in response to MITO was observed after STAT6 inhibition (2475 AU vs. 3285 AU, *p* < 0.05) (Fig. 5C). To test mitochondria-induced NETosis exclusivity, we conducted parallel testing and effect on NETs formation towards *S. aureus*. Inhibition of p38α and STAT6 did not reduce NETs formation (p38i: 2278 AU vs. 2098 AU, *p* > 0.05; STAT6i: 2759 AU, *p* > 0.05), while IL-4 pre-treatment significantly ameliorated NETs formation (2278 AU vs. 1502 AU, *p* < 0.05) (Fig. 5D).

## FPR1 agonism enhances mitochondrial phagocytosis and ROS production in response to* S. aureus*, while selectively impairing chemotaxis toward MITO in vitro

Having observed that FPR1 agonism selectively attenuated NET formation induced by MITO while maintaining the response to *S. aureus*, we investigated whether FPRA14 pre-treatment similarly affects other neutrophil effector functions, including reactive oxygen species (ROS) generation, phagocytosis, antimicrobial activity, and chemotaxis. ROS generation in response to MITO and *S. aureus* was analyzed after 30 min stimulation (Fig. 5E, F, S8 Fig). We have first confirmed that neutrophils have generated ROS in response to both MITO (1 → 1.94; *p* < 0.05) and *S. aureus* (1 → 3.26; *p* < 0.001). FPRA14 alone did not result in increased ROS burst in resting neutrophils (1 → 1.41; p > 0.05) and enhanced ROS production only in response to *S. aureus*, when compared to *S. aureus* treatment alone (3.26 → 4.41; *p* < 0.01; Fig. 5F). To determine whether FPRA14-mediated ROS enhancement depended on NADPH oxidase, we tested the selective NOX2 inhibitor GLX481304. Pharmacological NOX2 blockade completely abrogated enhanced ROS generation across all groups, reducing signal intensities to or below baseline control levels (Fig. 5F).

We have next assessed phagocytic uptake of MITO and *S. aureus*. Neutrophils displayed significantly higher phagocytic activity toward *S. aureus* than MITO at 10 min (50% vs. 18%; *p* < 0.001), with no effect of FPRA14 pre-treatment. By 30 min, MITO engulfment increased to 42% in untreated neutrophils and was further enhanced to 53% in FPRA14-pre-treated neutrophils (*p* < 0.05), while a 57% of *S. aureus* was phagocytized, again with no effect of FPRA14 pre-treatment (Fig. 5G). We observed that pre-treatment of neutrophils with FPRA14 did not alter their antimicrobial activity towards *S. aureus* when compared to untreated neutrophils (p > 0.05) (Fig. 5H).

To assess the effect on migratory behavior, we performed Transwell chemotaxis assays toward MITO, *S. aureus* and fMLF serving as a control in untreated and FPRA14-pre-treated neutrophils. As expected, fMLF induced robust neutrophil migration (55.88%; *p* < 0.001), with chemotactic responses toward MITO (30.55%; *p* < 0.01) and *S. aureus* (24.78%; *p* < 0.05) being lower. Notably, FPRA14 pre-treatment significantly suppressed chemotaxis towards fMLF (→ 24.54%; *p* < 0.01) and MITO (→ 8.59%; *p* < 0.01) but had no effect toward *S. aureus* (→ 22.16%; Fig. 5I). We additionally examined whether FPRA14 pre-treatment could modulate neutrophil chemotaxis toward *S. aureus* under conditions of concurrent MITO exposure. Neutrophils were pre-treated with FPRA14 (500 nM, 30 min) and/or MITO (15 min) in all pairwise combinations prior to chemotaxis assessment toward *S. aureus*. A rescue of chemotactic activity towards *S. aureus* was observed when MITO-exposed neutrophils were pre-treated with FPRA14 (12.96% vs. 27.05%; *p* < 0.05; Fig. 5J).

## Prophylactic FPRA14 dampens peritoneal NETosis while improving pulmonary bacterial clearance in vivo

**Fig. 6:**
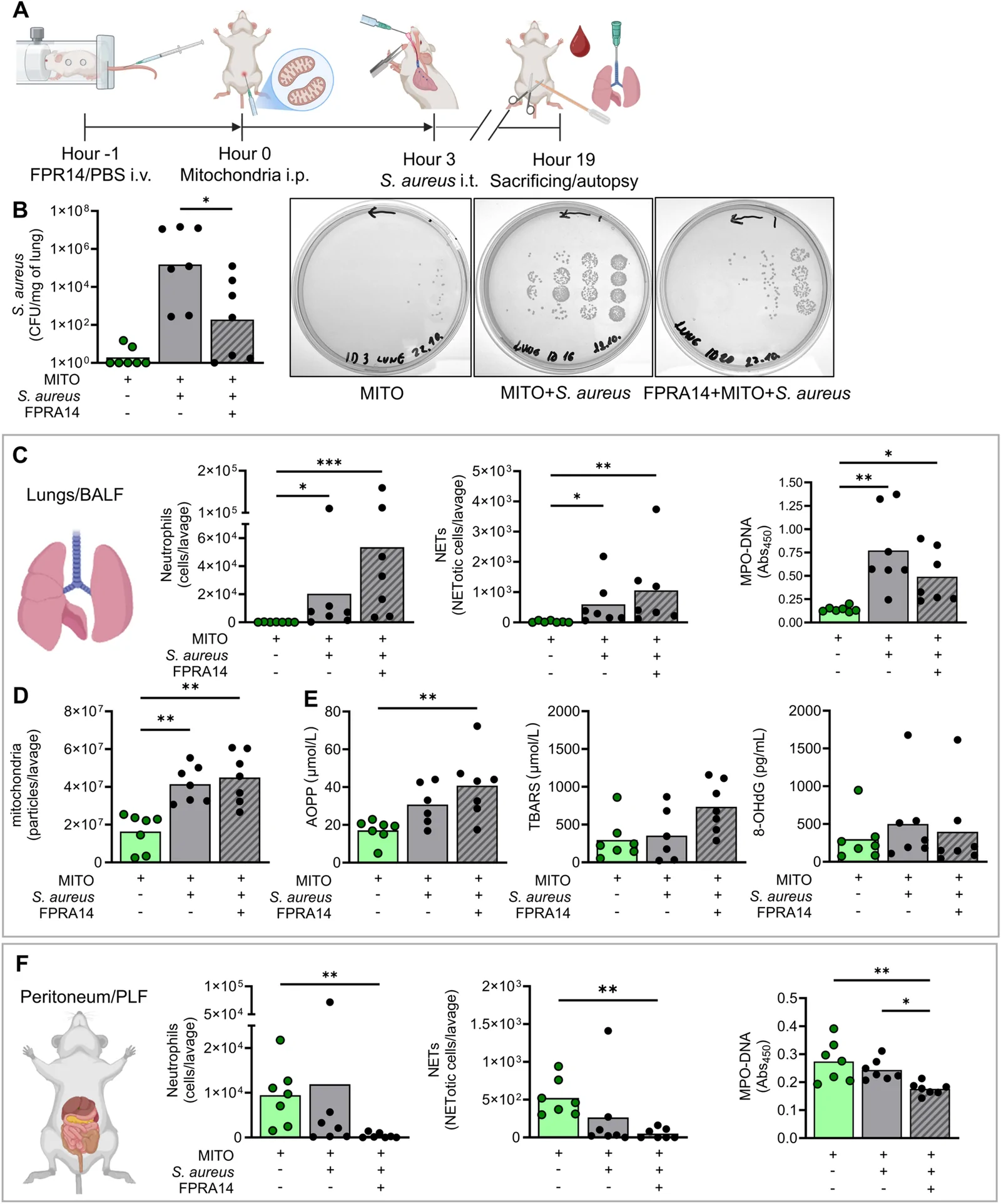
Prophylactic FPRA14 improves pulmonary bacterial clearance, attenuating neutrophil recruitment and NETs at peritoneal injuries. **A** Schematic overview of the experimental timeline. Mice received intravenous FPRA14 or PBS (hour − 1), intraperitoneal MITO (hour 0), intratracheal *S. aureus* (hour 3), and were sacrificed at hour 19 for tissue collection. **B** Left: *S. aureus* colony-forming units (CFU) per milligram of lung tissue across conditions (MITO only, MITO + *S. aureus*, FPRA14 + MITO + *S. aureus*). Right: Representative agar plates showing bacterial colony growth from lung homogenates. **C** Analysis of bronchoalveolar lavage fluid (BALF) from lungs. Left: total neutrophil count (cells/lavage). Middle: number of NETotic cells (NETotic cells/lavage). Right: MPO-DNA complex levels (Abs₄₅₀). **D** Quantification of MitoTracker-positive mitochondrial particles measured in BALF. **E** Quantification of oxidative lung damage in bronchoalveolar lavage fluid (BALF). Left panel: advanced oxidation protein products (AOPP). Middle panel: thiobarbituric acid reactive substances (TBARS). Right panel: 8-hydroxy-2'-deoxyguanosine (8-OHdG). **F** Analysis of peritoneal lavage fluid (PLF). Left: total neutrophil count (cells/lavage). Middle: number of NETotic cells (NETotic cells/lavage). Right: MPO-DNA complex levels (Abs₄₅₀). Data are presented as the mean with individual datapoints; *n* = 7 per group. *p < 0.05, **p < 0.01, ***p < 0.001; Kruskal–Wallis test with Dunn's post hoc test. Created in BioRender. Pastorek, M. (2026)

To test whether these findings translate in vivo, we employed a murine "double-hit" model of mitochondrial injury followed by secondary bacterial infection to mimic post-traumatic nosocomial pneumonia. Mice received intravenous FPRA14 or PBS (hour − 1), followed by intraperitoneal MITO administration (hour 0) and subsequent intratracheal *S. aureus* inoculation (hour 3), with sacrifice and tissue collection after 16 h (hour 19) (Fig. 6A). Mice receiving MITO followed by *S. aureus* displayed a substantial lung bacterial burden (5.5 × 10⁶ CFU/mg of lung; *p* < 0.05) with prophylactic FPRA14 administration significantly improving pulmonary bacterial clearance (1.5 × 10^5^ → 1.9 × 10^2^ CFU/mg; *p* < 0.05) (Fig. 6B).

To determine how FPRA14 shaped the neutrophil response at the site of infection, we analyzed bronchoalveolar lavage fluid (BALF) from infected lungs (Fig. 6C). Infection with *S. aureus* triggered neutrophil recruitment (2.03 × 10^4^ cells/lavage; *p* < 0.05, S3 Fig), and increased in FPRA14-pre-treated mice (5.35 × 10^4^ cells/lavage; *p* < 0.001). No significant difference was, however, observed in mice with *S. aureus* and *S. aureus* plus pre-treatment with FPRA14. The number of NETotic neutrophils in BALF was minimal in uninfected mice and significantly increased following *S. aureus* infection (6 × 10^2^ cells/lavage; *p* < 0.05), with no significant effect of FPRA14 pre-treatment (1 × 10^3^ cells/lavage). Similarly, MPO-DNA complexes in BALF were low in uninfected mice (Abs₄₅₀ = 0.14) and elevated after infection (Abs₄₅₀ = 0.77; *p* < 0.01) but not significantly altered after FPRA14 administration (Abs₄₅₀ = 0.49; Fig. 6C).

Given that FPRA14 prophylaxis promoted sustained neutrophil accumulation in the lungs, we assessed whether this resulted in collateral oxidative damage. Oxidative stress markers in bronchoalveolar lavage fluid were quantified: 8-hydroxy-2'-deoxyguanosine (8-OHdG; oxidative DNA damage), thiobarbituric acid reactive substances (TBARS; lipid peroxidation), and advanced oxidation protein products (AOPP), along with extracellular mitochondrial particles (Fig. 6D, E). Both *S. aureus*-infected groups showed markedly elevated extracellular mitochondrial particles in BALF compared to controls, independent of FPRA14 pre-treatment (1.64 × 10^7^ vs. 4.14 × 10^7^; 4.5 × 10^7^; *p* < 0.01, Fig. 6D). Protein oxidation (AOPP) was significantly higher in FPRA14-pre-treated mice versus uninfected controls (17.11 µmol/l vs. 40.78 µmol/L; *p* < 0.01, Fig. 6E). However, direct comparison between FPRA14-pre-treated and untreated *S. aureus*-infected mice revealed no significant differences in 8-OHdG, TBARS, or AOPP concentrations (Fig. 6E). In contrast, FPRA14 exerted a suppressive effect on the peritoneal neutrophil presence and NET burden (Fig. 6F). Neutrophil counts in peritoneal lavage fluid (PLF) were comparable between MITO-only (9.47 × 10^3^ cells/lavage) and MITO + *S. aureus* groups (11.89 × 10^3^ cells/lavage), but prophylactic FPRA14 markedly reduced peritoneal neutrophil infiltration (0.41 × 10^3^ cells/lavage; *p* < 0.01). FPRA14 administration also decreased the number of NETotic neutrophils in PLF (523 → 51 cells/lavage; *p* < 0.01), which was also reflected in reduced MPO-DNA complex levels (Abs₄₅₀ = 0.27 → 0.18; *p* < 0.01) (Fig. 6F).

In addition to microbial clearance and NET formation capacity, cytokine levels were profiled in both PLF and BALF (S9 Fig). In PLF, cytokine concentrations were largely unchanged across conditions, with the exception of IL-17a and IFN-β, which were decreased in the FPRA14 + MITO + *S. aureus* group compared to MITO alone (*p* < 0.01 and *p* < 0.05, respectively; S9 Fig). In BALF, *S. aureus* infection was associated with a broad increase in cytokine concentrations (Supplementary Fig. 6B). IL-1α (*p* < 0.01), MCP-1 (*p* < 0.01), GM-CSF (*p* < 0.001), IL-12p70 (*p* < 0.05), IFN-β (*p* < 0.05), IFN-γ (*p* < 0.01), IL-17a (*p* < 0.05), IL-10 (*p* < 0.01), and IL-27 (*p* < 0.001) were significantly elevated in both the MITO + *S. aureus* and FPRA14 + MITO + S. aureus groups compared to MITO alone. IL-1β and TNF-α were elevated exclusively in the FPRA14 + MITO + *S. aureus* group compared to MITO alone (both *p* < 0.05). No significant differences in any measured cytokine were observed between the MITO + *S. aureus* and FPRA14 + MITO + *S. aureus* groups in either compartment.

## Discussion

Mitochondrial DAMPs correlate with post-traumatic complications and mortality (Simmons et al. 2013; Zhang et al. 2010) and, as we have previously shown, drive NET formation in vivo (Pastorek et al. 2023). What has been lacking is a mechanistic framework for how this response is regulated and whether it can be therapeutically dissociated from antimicrobial NETosis. Here, we demonstrate that intact mitochondria induce NETosis through coordinated TLR9–FPR1 engagement with PAD4 and NOX2 dependence. Furthermore, FPR1 agonism via FPRA14 selectively suppresses response to mtDAMPs while fully preserving or even enhancing antibacterial NET formation, phagocytosis, ROS production and bactericidal killing in vitro, ex vivo in trauma patient plasma, and in vivo in a dual-hit model of surgical injury followed by bacterial pneumonia.

We first establish that laparotomy releases mitochondria into the circulation, coinciding with robust neutrophil recruitment and NET formation by 4 h. Exogenous administration of isolated mitochondria recapitulated this response, extending prior work that either demonstrated mtDAMP-driven neutrophil activation (ROS, degranulation, IL-8) or detected CitH3 in trauma patients within minutes of injury, but did not attribute NETosis specifically to extracellular mitochondria (Hazeldine et al. 2015; Hampson et al. 2017; Briggs et al. 2025). As previously noted, most mechanistic studies employ individual purified mtDAMPs (mtDNA, ATP, succinate, etc.) as stimuli, yet neutrophils in vivo encounter whole or partially degraded organelles presenting multiple DAMPs simultaneously (Bečka et al. 2026). Our component dissection reveals why this matters: isolated mtDNA (up to 5 µg/ml) was a poor NET inducer and a weak TLR9 activator, whereas whole mitochondria were substantially more potent in both respects. Pre-treating mitochondria with DNase had no effect on NET induction, whereas protein depletion by proteinase-K completely abrogated it. This does not suggest limited role for mtDNA in NET formation, but clarifies that efficient NET induction requires either supraphysiological mtDNA concentrations or protein-mediated protection from degradation, potentially in synergy with other mitochondrial components (Itagaki et al. 2015). These findings are consistent with prior reports that mtDNA alone fails to activate p38 MAPK signaling in neutrophils, and that formylated peptides potentiate the limited activation mtDNA achieves (Zhang et al. 2010; Hazeldine et al. 2015; Sun et al. 2013; Prikhodko et al. 2015). Importantly, targeting TLR9, NOX2, PAD4, or FPR1 individually each only partially inhibited MITO-driven NETosis, indicating a multi-pathway signaling architecture. This provides a mechanistic basis for the contextual NOX-dependence of mtDAMP-induced NET formation observed in prior studies and aligns with the emerging view that NETosis requires the convergence of multiple simultaneous activating signals (Itagaki et al. 2015; Alarcón et al. 2020; Karmakar et al. 2016; Sofoluwe et al. 2019; Zukas et al. 2024).

This does not preclude investigation of the NET-inducing capacity of individual mitochondrial components, but rather underscores the importance of examining their interactions, as exemplified by the controversial role of formylated peptides in NETosis (Kim et al. 2020; Li et al. 2023; Zhu et al. 2018; Ohbuchi et al. 2017). While studies from diverse inflammatory clinical context implicate FPR1 in NET formation (Li et al. 2025; Zhang et al. 2025; Lefrançais et al. 2018), mechanistic data on the NET-inducing capacity of formylated peptides range from no induction to induction at nanomolar concentrations (Carvalho Oliveira et al. 2023; Kuhikar et al. 2020; Mol et al 2021; Tatsiy et al. 2018; Tatsiy and McDonald 2018). Our data reframe FPR1 not as a primary inducer but as a concentration- and time-dependent regulator of NETosis. We observed that established bacterial or synthetic-origin FPR1 agonists (fMLF, FPRA14) had limited capacity to trigger NET formation alone, yet both pharmacological antagonism and agonism of FPR1 significantly reduced MITO-driven NETosis. At moderate agonist concentrations and with concurrent treatment, FPR1 synergizes with TLR9 engagement to promote NETosis; at high concentrations or with sustained pre-exposure, the response shifts toward attenuation. This biphasic behavior is mechanistically consistent with the previously demonstrated concentration-dependent biased signaling of FPR1 and its selective activation of distinct phagocyte functions (Wang and Ye 2022). In support of this, relative surface expression of FPR1 was increased after 20 min of mitochondrial stimulation, and FPRA14 pre-treatment normalized this upregulation. The functional consequence of this FPR1 regulation was further demonstrated using trauma patient plasma. While trauma plasma contains a complex array of systemic mediators, the finding that FPRA14 pre-treatment significantly attenuated plasma-induced NETosis confirms that this response is predominantly FPR1-dependent. This is conceptually complementary to the observation that clearance of circulating mitochondrial N-formyl peptides from septic patient plasma restores neutrophil function (Kwon et al. 2024).

While that study focused on removing the agonist to prevent FPR1 engagement, we reasoned that the converse strategy, exploiting controlled FPR1 desensitization as a preemptive intervention, may also be used as prophylaxis before anticipated tissue damage. This approach is particularly viable in controlled clinical settings of "planned trauma," such as major elective surgeries (e.g., cardiac surgery utilizing cardiopulmonary bypass or extensive oncological resections). In these scheduled scenarios, the timing of tissue injury and the subsequent systemic surge of mtDAMPs are predictable, establishing a definitive therapeutic window for prophylactic receptor modulation. Perhaps the most significant finding in this regard is that FPRA14-mediated FPR1 desensitization selectively suppressed mitochondria-driven NETosis while fully preserving *S. aureus*-induced NET formation, phagocytosis, and bactericidal killing. This selectivity extends to *E. coli* (S7 Fig), indicating that the phenomenon is not restricted to a single pathogen species or Gram type. Canonically, FPR1 signals through Gαi-mediated PLCβ activation and ER Ca^2^⁺ release (O'Flaherty et al. 1990). However, neither mitochondria nor *S. aureus* induced significant SOCE mobilization compared to fMLF, and extracellular Ca^2^⁺ chelation with EGTA did not affect mitochondria-driven NETosis. FPR1's contribution to this response thus likely operates through a Ca^2^⁺-independent pathway. Consistently, FPRA14 pre-treatment did not alter fMLF-induced Ca^2^⁺ flux.

This prompted phosphokinase profiling, which fundamentally different signaling architectures downstream of MITO and *S. aureus* stimulation. *S. aureus* induced broad phosphorylation across nearly all kinases assayed, consistent with the multi-receptor engagement expected from a whole pathogen. By contrast, neutrophils stimulated by mitochondria had a more restricted signature and selectively activated p38α, p70 S6K, STAT6, and β-Catenin, consistent with a limited receptor repertoire (principally FPR1 and TLR9) for mitochondria-induced NET formation. This may explain why desensitizing a single receptor (FPR1) is sufficient to attenuate mitochondria- but not pathogen-driven NETosis: bacteria engage a far more redundant receptor network that compensates for FPR1 loss. Beyond NETosis, FPRA14 pre-treatment selectively impaired chemotaxis toward mitochondria and fMLF without significantly affecting migration toward *S. aureus*, and enhanced mitochondrial phagocytosis at 30 min while leaving *S. aureus* phagocytosis unchanged. Notably, FPRA14 also rescued *S. aureus* chemotaxis that had been impaired by concurrent mitochondrial exposure, suggesting that FPR1 desensitization may actively protect antimicrobial neutrophil function from interference by mitochondria, consistent with previous findings by group of Dr. Hauser (Itagaki et al. 2020; Kwon et al. 2024; Li et al. 2015). Notably, FPRA14 pre-treatment enhanced intracellular ROS generation in response to *S. aureus* but not to mitochondria. We hypothesize that FPR1 desensitization reduces the cumulative signaling strength driving mitochondria-induced NETosis without compromising the redundant pathways activated by *S. aureus*. Alternative mitochondrial recognition pathways (TLR9 and purinergic receptors) may then remain intact and maintain baseline ROS production, but without the specific co-signals from FPR1 may be insufficient to drive NETosis in this context.

The identification of p38α as a mediator of mitochondria-induced NETosis extends the findings of Hazeldine et al., who demonstrated that p38 and ERK1/2 MAPK activation is essential for mtDAMP-induced neutrophil activation in a FPR1-dependent manner (Hazeldine et al. 2015). We now show that p38α inhibition attenuates mitochondria- but not *S. aureus*-induced NET formation, indicating that whereas p38 is an important signaling node in mitochondria- but not *S. aureus*-induced NETosis. STAT6 emerged as a novel negative regulator of mitochondria-induced NETosis: its phosphorylation was markedly upregulated by mitochondria and STAT6 activation with IL-4 suppressed NET formation while STAT6 inhibition enhanced it. Together with the parallel suppression of p38α, this may partly account for the anti-NETotic effects of FPR1 desensitization. In myeloid cells, STAT6 signaling has been proposed as a homeostatic mechanism to limit excessive inflammation and tissue damage, so our results are consistent with its anti-inflammatory and pro-resolving roles in sterile and endotoxin-driven inflammation (Wermeling et al. 2013; Kaplan et al. 1996; Karpathiou et al. 2021; Lee et al. 2021; Lentsch et al. 2001). The role of STAT6 in NET biology remains poorly defined. Currently limited to a single study in cerebral amyloid angiopathy, NET-induced STAT6 activation was reported to perpetuate NET formation, which is in contrast to the suppressive role we observe (Huang et al. 2024). Whether this discrepancy reflects context-dependent signaling is unknown, but it underscores the need for further investigation of STAT6 as a NETosis regulator.

To evaluate these findings in a physiologically relevant context, we employed a dual-hit model of mitochondrial injury followed by bacterial pneumonia. Prophylactic FPRA14 administration reduced peritoneal neutrophil recruitment, NETotic cell numbers and MPO-DNA complexes (NETs) at the sterile injury site. Most importantly, pulmonary neutrophil recruitment and NET formation was maintained, with bacterial clearance improved by over three orders of magnitude compared to untreated controls. Cytokine profiles did not differ between FPRA14-treated and untreated mice in either compartment, arguing against global immunomodulation. This is noteworthy in context of prior findings where DAMP (specifically formylated peptide) exposure induced functional tolerance that impaired subsequent antimicrobial responses (Kaczmarek et al. 2018; Hazeldine et al. 2019; Kwon et al. 2021). The improved bacterial clearance in FPRA14-treated mice may reflect the preservation of neutrophil function that would otherwise be exhausted by the ongoing sterile inflammatory response. To evaluate whether enhanced neutrophil recruitment in FPRA14-treated mice would increase oxidative damage to lung tissue, we assessed protein oxidation, lipid peroxidation, and DNA oxidative damage in pulmonary tissue from *S. aureus*-infected mice. Compared to untreated controls, FPRA14-treated animals showed no significant increase in protein or DNA oxidation, though a non-significant trend toward increased lipid peroxidation was observed.

These results suggest that enhanced neutrophil presence and ROS production in FPRA14-treated lungs do not translate into substantial collateral tissue damage under the conditions tested. However, we acknowledge that the time-course of neutrophil residence in lung tissue and the duration of ROS production after FPRA14 pre-treatment are incompletely characterized. Future studies with extended observation periods and resolution assays examining neutrophil clearance and tissue repair kinetics will be necessary to determine whether acutely enhanced bacterial clearance provides a net protective benefit to lung tissue.

Several limitations of this study should be acknowledged. FPR1 desensitization is inherently reversible; full receptor recovery has been reported within 30 min of agonist removal, with complete functional restoration by 90 min (Kwon et al. 2024). Optimal dosing, timing, and the potential need for repeated administration in a clinical prophylaxis setting therefore require further investigation. Selectivity has been confirmed against both *S. aureus* (Gram-positive) and *E. coli* (Gram-negative), but whether it is preserved across a broader pathogen spectrum remains unknown. Finally, neutrophil heterogeneity influenced by maturation state and density, both relevant factors during emergency granulopoiesis after trauma, may modulate susceptibility to FPRA14.

## Conclusions

We provide the first systematic characterization of how mitochondria drive NETosis through coordinated multi-receptor signaling and demonstrate that FPR1 operates as a concentration- and time-dependent regulator of this response. Exploiting FPR1 desensitization with FPRA14 selectively dissociates pathological sterile NETosis from antimicrobial immunity at the receptor level. Prophylactic FPR1 agonism before anticipated tissue damage therefore represents a conceptually distinct strategy to attenuate post-operative sterile inflammation while preserving host defense.

## Supplementary Information


Supplementary Material 1.


## Data Availability

All data generated or analysed during this study are included in this published article [and its supplementary information files].

## Abbreviations

BALF: Bronchoalveolar lavage fluid
*E. **coli*: *Escherichia* *coli*
ER: Endoplasmic reticulum
fMLF: N-formylmethionine-leucine-phenylalanine
FPR1: Formyl peptide receptor 1
MAPK: Mitogen-activated protein kinases
MITO: Mitochondria
MPO: Myeloperoxidase
mtDAMPs: Mitochondrial damage–associated molecular patterns
mtDNA: Mitochondrial DNA
ncDNA: Nuclear DNA
NET: Neutrophil extracellular traps
NOX2: NADPH oxidase 2
ODN: Oligodeoxynucleotides
PAD4: Peptidyl arginine deiminase 4
PLF: Peritoneal lavage fluid
ROS: Reactive oxygen species
RT: Room temperature
*S. **aureus*: *Staphylococcus* *aureus*
OCE: Store-operated Ca2^+^ entry
SURG: Surgery-related trauma
TLR9: Toll-like receptor 9
