# Supplementary material for: Formyl peptide receptor 1 agonism selectively suppresses mitochondria-driven neutrophil extracellular trap formation while preserving antibacterial immunity
Source: Mol Med. 2026 Jun 13;32:137. doi: 10.1186/s10020-026-01522-4 (PMC13523471; doi:10.1186/s10020-026-01522-4)
Supplement: Supplementary file 1 [file 10020_2026_1522_MOESM1_ESM.pdf]

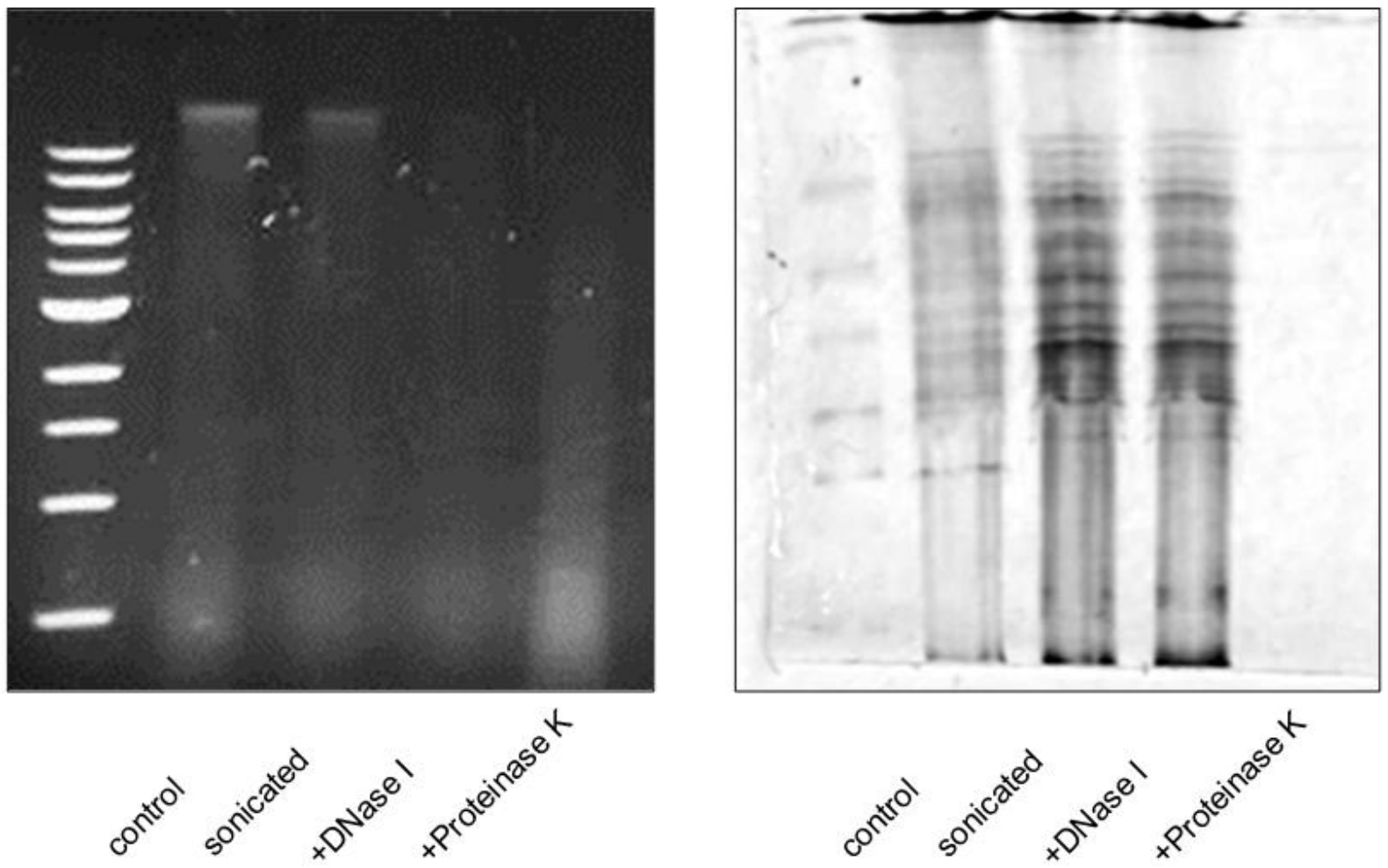

S1 Fig. Mitochondria digestion

Representative 1.5% agarose (left) and 10% SDS-PAGE (right) gels confirming degradation of mitochondrial DNA and proteins, respectively, in mitochondrial samples after enzymatic processing to deplete mtDNA (+DNase I) and proteins (Proteinase K).

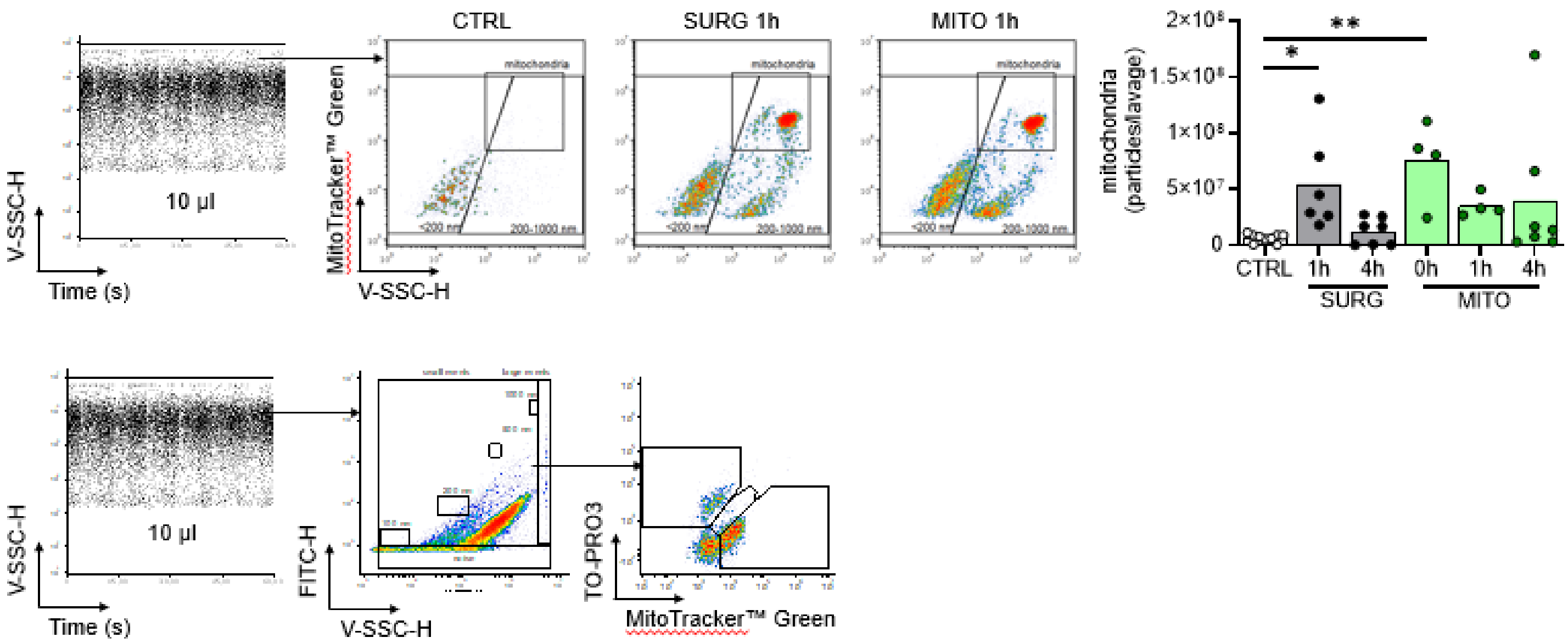

S2 Fig. Mitochondria release after surgery

Representative flow cytometry plots (top left) and quantification (right) of muse mitochondrial particles in peritoneal lavage fluid, identified by MitoTracker™ Green staining, at 1h and 4h post-surgery (SURG) or 0h, 1h, and 4h post-injection (MITO). Representative flow cytometry plots of mitochondrial particles (bottom) in human plasma identified by MitoTracker™ Green and TO-PRO-3 co-staining. Data are presented as the mean with individual datapoints; n = 6 per group. *$p<0.05$, **$p<0.01$, ***$p<0.001$ vs. CTRL; one-way ANOVA with Dunnet's post hoc test.

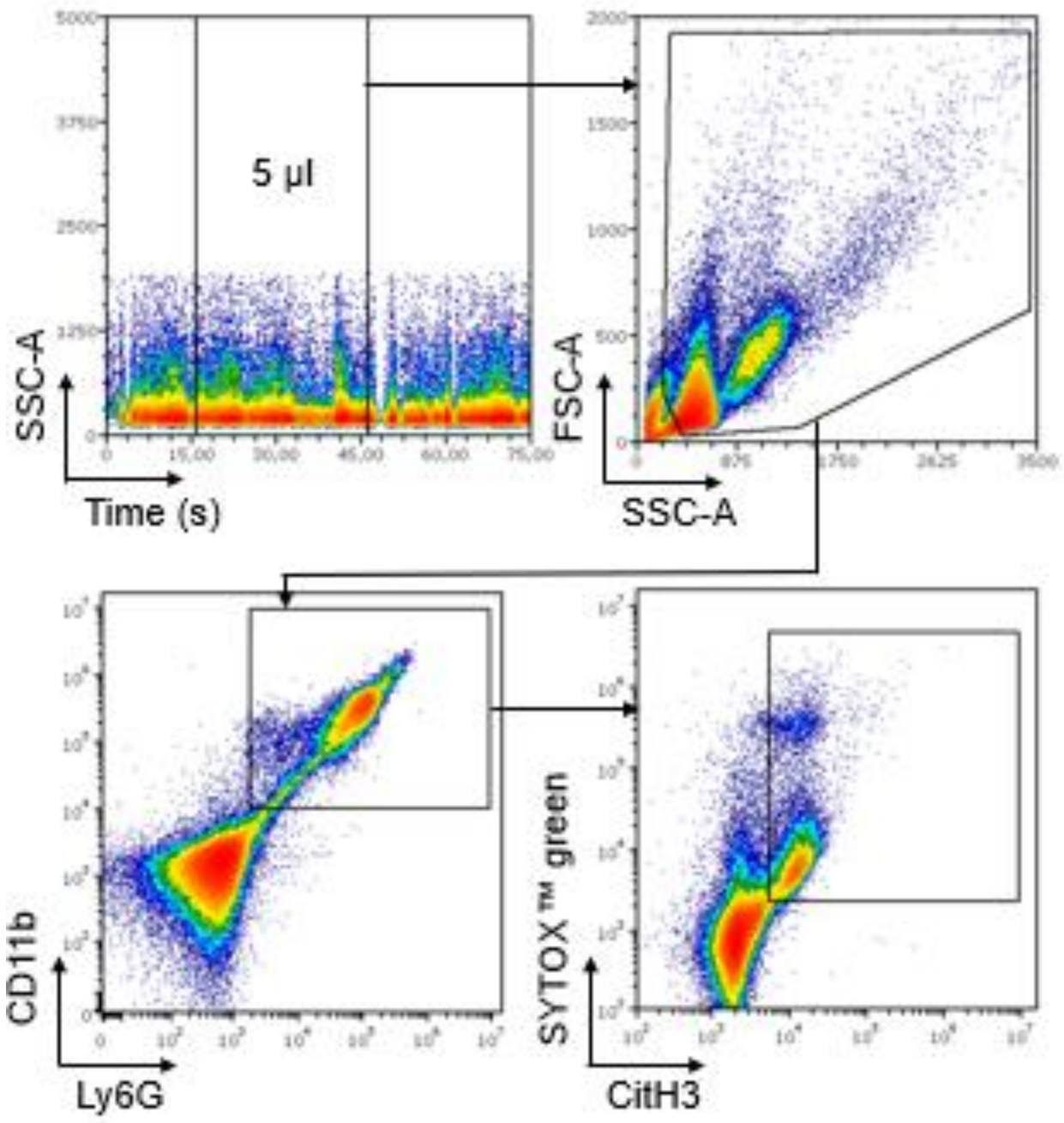

S3 Fig. Neutrophils and NETotic cells gating strategy

Gating strategy for the analysis of neutrophil swarming and NETotic cells in mouse bronchoalveolar lavage fluid (BALF) and peritoneal lavage fluid (PLF). Neutrophils were identified as CD11b and Ly6G double-positive events. Within this population, cells positive for both SYTOX™ Green and citrullinated histone 3 (citH3) were defined as NETotic neutrophils. Both total and NETotic neutrophils were quantified according to volume of the sample aspirated with pre-defined flow rate and recalculated to total BALF volume.

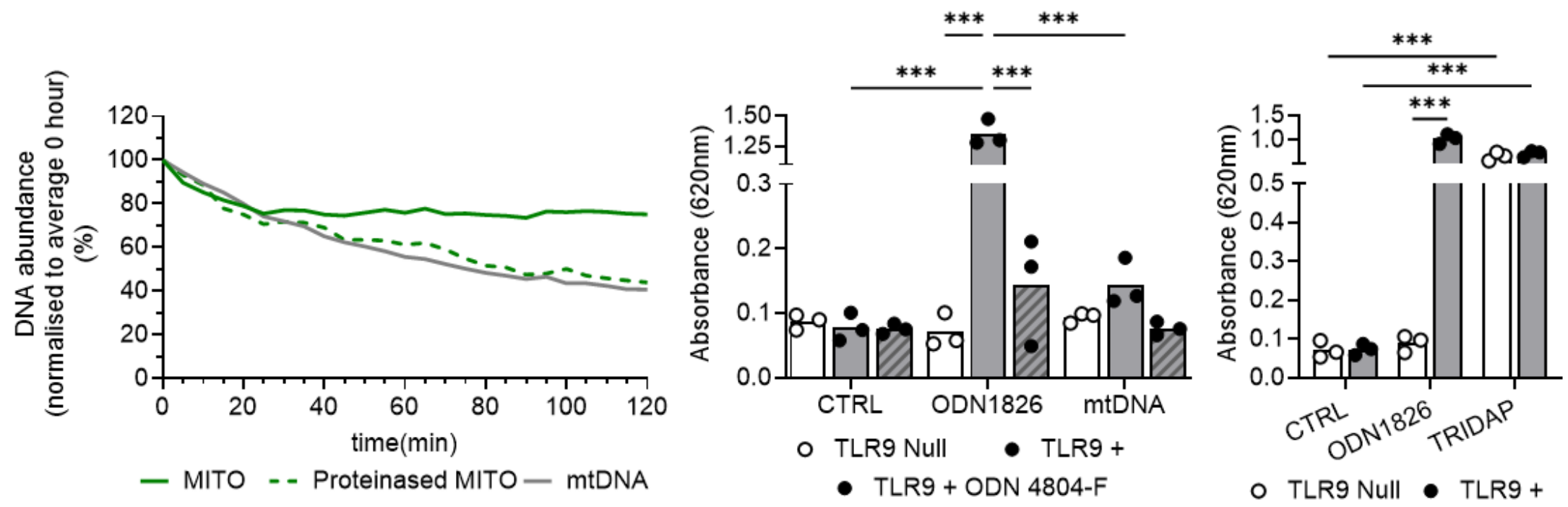

S4 Fig. Protected mtDNA is stable and exhibits higher immunogenicity

(Left) mtDNA degradation kinetics (% from the input abundance) over 120 minutes. (middle) and PMA (100 nM). (Middle) NF-κB reporter activation (absorbance at 620 nm) in TLR9-expressing (TLR9+) and TLR9-null reporter cells pre-treated with inhibition oligo ODN 4804-F (50µg/ml) following stimulation with oligo ODN1926 (50µg/ml), mtDNA (50µg/ml). (Right) NF-κB reporter activation (absorbance at 620 nm) in TLR9+ vs TLR9− reporter cells, with positive control and TRIDAP (NOD1 agonist) exposure as assay control. Data are presented as the mean with individual datapoints; ***p<0.001 two-way ANOVA with Šidák's multiple comparison test.

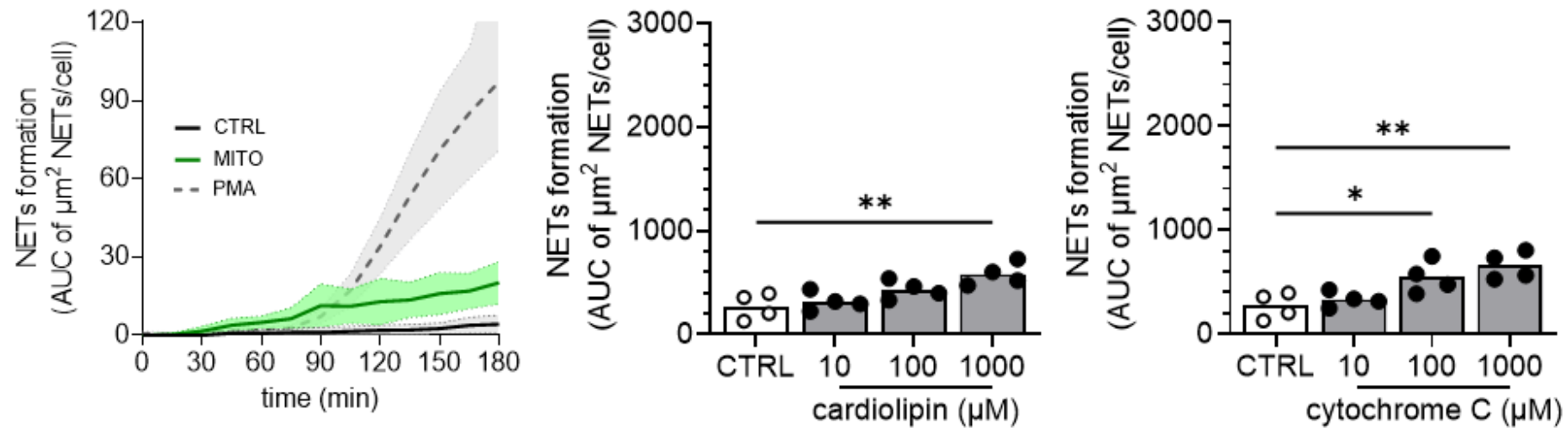

S5 Fig. Mitochondrial cardiolipin and cytochrome C are insufficient to induce robust NETs formation

(Left) NET formation kinetics (µm²/cell) over 180 minutes in neutrophils co-incubated with intact mitochondria (MITO; 1×10^7 particles) and PMA (100 nM). (Middle) Total NET formation quantified as AUC (µm² NETs/cell) for neutrophils co-incubated with cardiolipin and (right) cytochrome C. Data are presented as the mean with individual datapoints; n = 4. *$p<0.05$, **$p<0.01$, CTRL; one-way ANOVA with Dunnet's post hoc test.

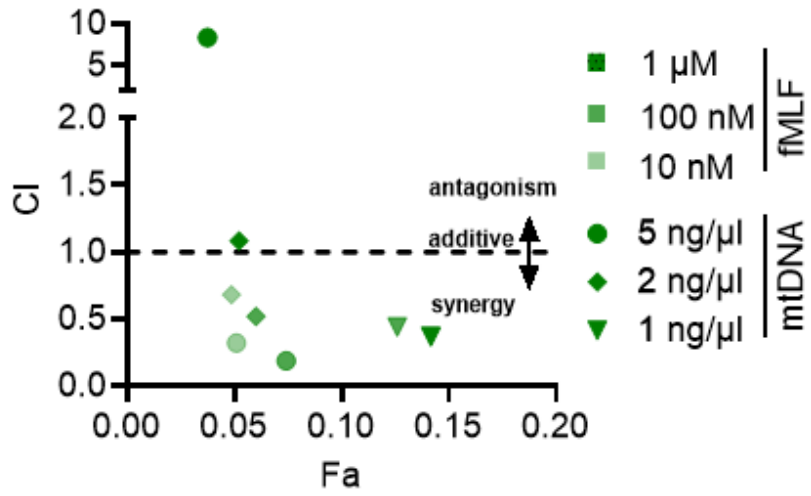

S6 Fig. Synergistic induction of NETs formation by formylated peptide and mtDNA

Chou-Talalay combination index (CI) analysis of mtDNA and fMLF interactions across a concentration matrix. CI <1 indicates synergy; CI >1 indicates antagonism; CI = 1 indicates additivity.

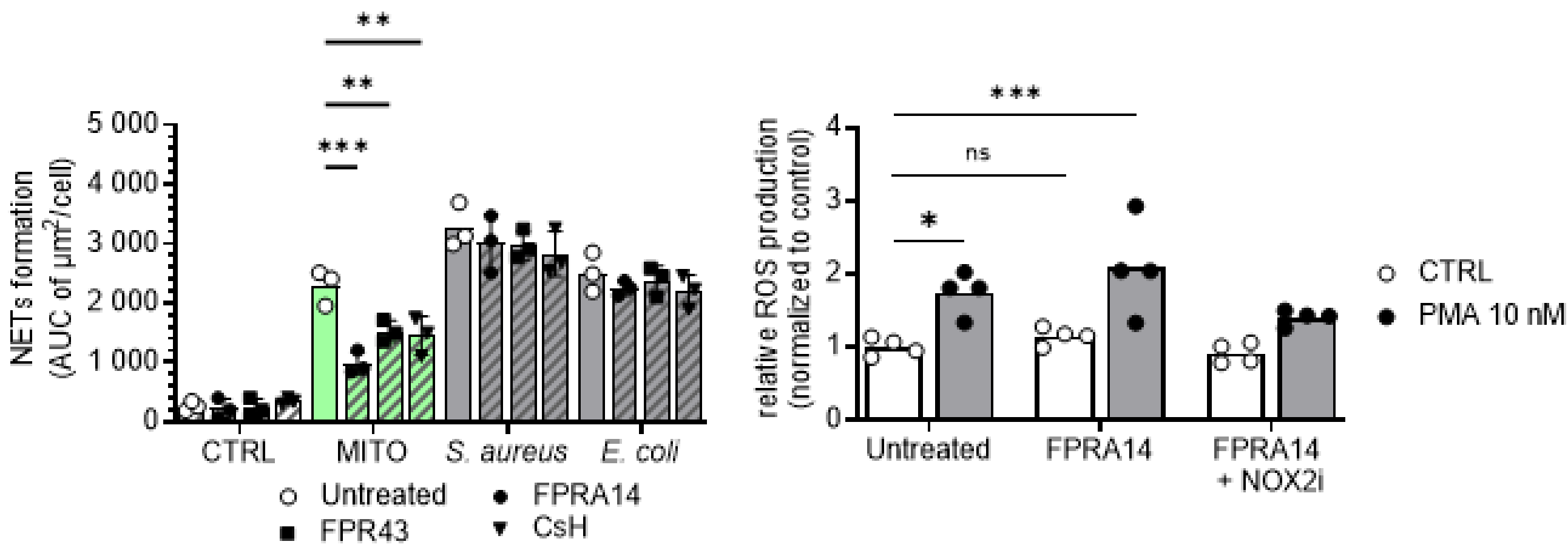

S7 Fig. Selective FPR1 modulation decreases MITO-triggered NETs formation and does not modulate ROS production

(Left) Total NET formation (AUC of µm² NETs/cell) following co-incubation with MITO, *S. aureus*, *E. coli* in untreated or FPRA14 (500 nM), FPR43 (500 nM), and Cyclosporin H (CsH; 10 µM)-pre-treated neutrophils. Data are presented as the mean with individual datapoints **p<0.01, ***p<0.001; two-way ANOVA with Šidák's multiple comparison test. (Right) Relative intracellular reactive oxygen species (ROS) production in neutrophils pre-treated with FPRA14 (500 nM), and GLX481304 (NOX2i; 2.5 µM) and stimulated with 10 nM PMA, normalized to control untreated neutrophils. *p<0.05, ***p<0.001; two-way ANOVA with Šidák's multiple comparison test.

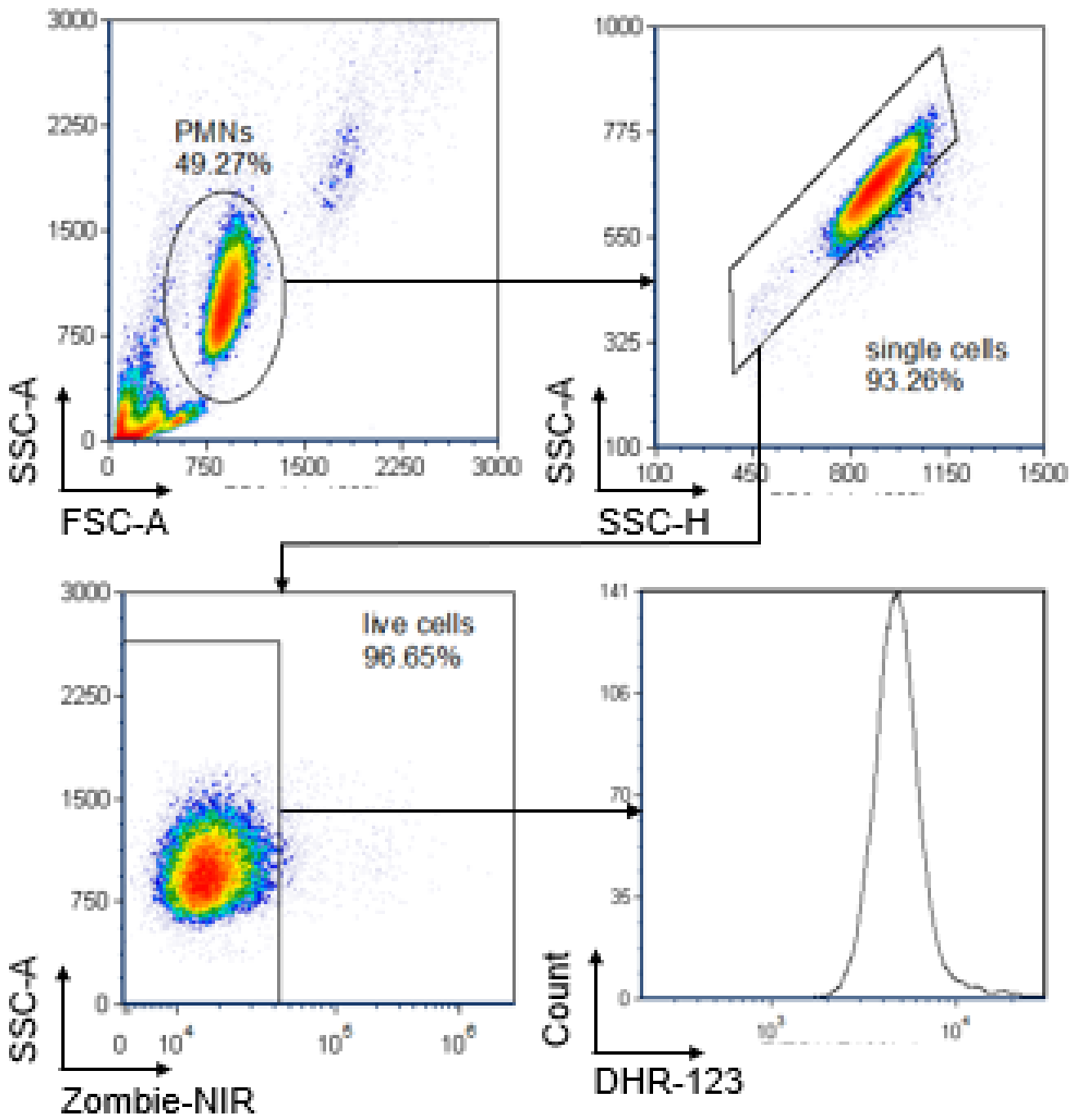

S8 Fig. Neutrophils ROS quantification gating strategy

Gating strategy for the analysis of intracellular ROS production in human neutrophils. Neutrophils were identified based on size and granularity using forward and side scatter. Subsequently, viable (Zombie NIR-negative) cells were gated to analyze the Dihydrorhodamine 123 (DHR123) signal. ROS production was quantified from the mean fluorescence intensity (MFI) of samples after background substraction, normalized to average MFI of control samples.

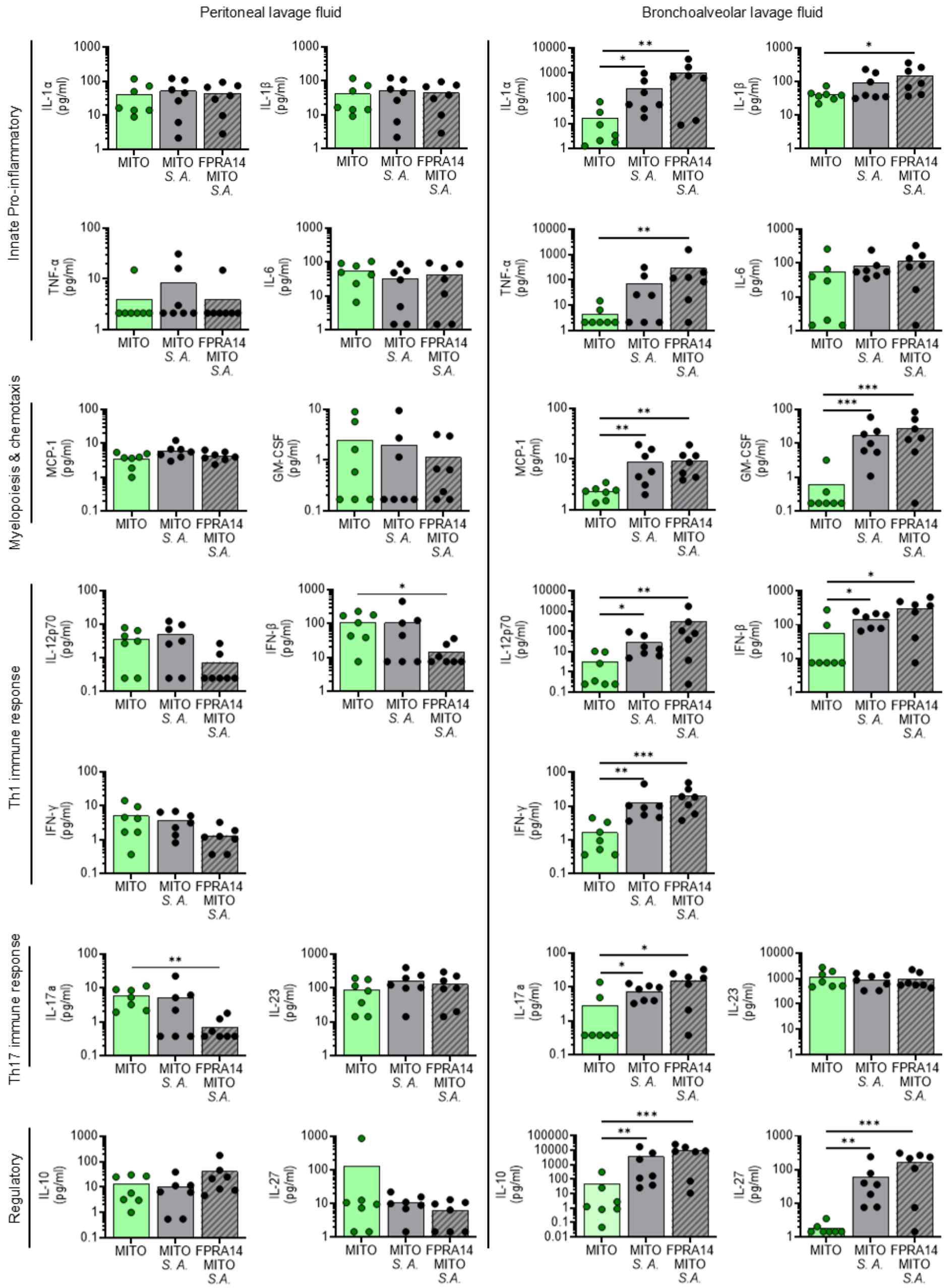

S9 Fig. Inflammatory cytokine concentrations in peritoneal lavage fluid and bronchoalveolar lavage fluid

Inflammatory cytokines in (left) peritoneal lavage fluid and (right) bronchoalveolar lavages. IL-1α- interleukin 1-alpha; IL-1β- interleukin 1-beta; TNF-α- tumor necrosis factor alpha; IL-6- interleukin 6; IL-10- interleukin; MCP-1- monocyte chemoattractant protein-1; IL-17A- interleukin 17A; IL-23- interleukin 23; interleukin 27 (IL-27) concentrations in males; INF-β- interferon beta; INF-γ- interferon gamma GM-CSF- granulocyte-macrophage colony-stimulating factor; IL-12p70- interleukin 12p70. Data are presented as the mean with individual datapoints; n = 7 per group. *p<0.05, **p<0.01, ***p<0.001; *p<0.05, **p<0.01, **p<0.001 vs. CTRL; one-way ANOVA with Dunnet's post hoc test.
